# A new tumorgraft panel to accelerate precision medicine in prostate cancer

**DOI:** 10.3389/fonc.2023.1130048

**Published:** 2023-05-26

**Authors:** Claire Béraud, Nadege Bidan, Myriam Lassalle, Hervé Lang, Véronique Lindner, Clémentine Krucker, Julien Masliah-Planchon, Eric Potiron, Philippe Lluel, Thierry Massfelder, Yves Allory, Yolande Misseri

**Affiliations:** ^1^ Urosphere, Toulouse, France; ^2^ Department of Urology, Nouvel Hopital Civil, Strasbourg, France; ^3^ Department of Pathology, Hôpital de Hautepierre, Strasbourg, France; ^4^ Department of Pathology, Institut Curie, Paris, France; ^5^ Institut Curie, PSL Research University, CNRS, Equipe Labellisée Ligue Contre le Cancer, Paris, France; ^6^ Department of Genetics, Institut Curie, Paris, France; ^7^ Department of Urology, Clinique Urologique, Nantes, France; ^8^ UMR 1260 INSERM/Université de Strasbourg, Regenerative Nanomedicine (RNM), FMTS, Centre de Recherche en Biomédecine de Strasbourg, Strasbourg, France

**Keywords:** PDX, prostate cancer, neuroendocine tumors, genomic characteristics, PARP inhibitor, metabolism, tumor heterogeneity, castrate-resistant prostate cancer (CRPC)

## Abstract

**Background:**

Despite the significant advances in the management of advanced prostate cancer (PCa), metastatic PCa is currently considered incurable. For further investigations in precision treatment, the development of preclinical models representing the complex prostate tumor heterogeneity are mandatory. Accordingly, we aimed to establish a resource of patient-derived xenograft (PDX) models that exemplify each phase of this multistage disease for accurate and rapid evaluation of candidate therapies.

**Methods:**

Fresh tumor samples along with normal corresponding tissues were obtained directly from patients at surgery. To ensure that the established models reproduce the main features of patient’s tumor, both PDX tumors at multiple passages and patient’s primary tumors, were processed for histological characteristics. STR profile analyses were also performed to confirm patient identity. Finally, the responses of the PDX models to androgen deprivation, PARP inhibitors and chemotherapy were also evaluated.

**Results:**

In this study, we described the development and characterization of 5 new PDX models of PCa. Within this collection, hormone-naïve, androgen-sensitive and castration-resistant (CRPC) primary tumors as well as prostate carcinoma with neuroendocrine differentiation (CRPC-NE) were represented. Interestingly, the comprehensive genomic characterization of the models identified recurrent cancer driver alterations in androgen signaling, DNA repair and PI3K, among others. Results were supported by expression patterns highlighting new potential targets among gene drivers and the metabolic pathway. In addition, *in vivo* results showed heterogeneity of response to androgen deprivation and chemotherapy, like the responses of patients to these treatments. Importantly, the neuroendocrine model has been shown to be responsive to PARP inhibitor.

**Conclusion:**

We have developed a biobank of 5 PDX models from hormone-naïve, androgen-sensitive to CRPC primary tumors and CRPC-NE. Increased copy-number alterations and accumulation of mutations within cancer driver genes as well as the metabolism shift are consistent with the increased resistance mechanisms to treatment. The pharmacological characterization suggested that the CRPC-NE could benefit from the PARP inhibitor treatment. Given the difficulties in developing such models, this relevant panel of PDX models of PCa will provide the scientific community with an additional resource for the further development of PDAC research.

## Introduction

Prostate Cancer (PCa) is the second most frequent cancer in men and the fifth leading cause of cancer death, with an incidence rate of 14.3% (1). Different molecular subtypes of PCa have been determined according to their genomic alterations. They have been classified from localized early stage to advanced/metastatic tumors. Localized/primary PCa generally demonstrate few genomic alterations and are sensitive to androgen deprivation therapies (ADT). Castrate-Resistant Prostate Cancers (CRPC) and metastatic prostate cancers mPCa demonstrate an increase in number and severity of genomic alterations and become insensitive to ADT (2). Locally confined PCa can be treated effectively, as first line therapy either, by surgical resection or radiation therapy (3). For non-organ-confined tumors, the standard treatment is medical or surgical castration. Androgen Receptor (AR) overexpression is a main driver of progression to CRPC for most patients (4). Androgen deprivation is an effective therapeutic strategy, widely used in clinical practice. These treatments include potent new generation hormonotherapy such as abiraterone acetate and enzalutamide, that has improved patient outcomes. However, most patients relapse within 2-3 years after initial response and the disease progresses to CRPC (5–7). Metastatic CRPC (mCRPC) is a heterogeneous disease with poor outcomes. In up to 30% of patients, tumors harbour deleterious aberrations in the genes involved in repairing DNA damage. Pembrolizumab, an immune checkpoint inhibitor, demonstrates a high response rate in tumors with mismatch repair deficiency regardless of primary site (8), leading to tissue-agnostic FDA approval, including for PCa (9). Pharmacological inhibitors of poly (ADP-ribose) polymerase (PARP) have been recently approved for use in patients with advanced PCa harbouring homologous recombination defects, including *BRCA1* and *BRCA2* alterations (10). These mutations are not present in all PCa, and despite these new clinical developments, PCa remains incurable when these therapies fail. Further new preclinical models and studies should thus explore mechanisms of resistance based on clinical data and available experimental models.

Patient-Derived-Xenografts (PDXs) are based on the direct implantation of fresh cancer tissue specimens from individual patients into immunodeficient mice or rats (11). Their development has been optimized over time concomitantly with the discovery of and advancements in immunocompromised animal models. PDXs have the advantage of retaining the cellular heterogeneity, architecture, and molecular characteristics of the parental tumor (12, 13). Numerous studies have cited them as the best predictors of response compared to cell line-derived xenografts which, with time, lose heterogeneity and tend to be clonally selected (14). Efforts to develop xenografts from PCa have been made since the 1970’s with varying degrees of success due to the particular difficulty of developing these models. Reported take rate in established PDX from PCa range between 0 to 33%, while longest spanning latencies vary from 60 to 1,147 days (15). Despite these drawbacks and due to the heterogeneity of the disease and complexity, PCa PDX models remain the most accurate preclinical models for biological studies, drug development and personalized medicine strategies (16–20).

As a result of a 10-year research project in urological PDX models, the present work describes a prostate PDX biobank of 5 established PDXs models (out of 240 PCa originally implanted). The developed panel of PDXs recapitulate the progression of the disease from androgen sensitive to CRPC, including CRPC with neuroendocrine (NE) features. In addition, from the same patient that became AR-resistant after treatment, an AR-sensitive adenocarcinoma and an AR-resistant neuroendocrine PDXs have been derived and characterized.

## Materials and methods

### Acquisition of PCa patient tissues

Between 2010 and 2019, thanks to a close collaboration with the Pathology and Urology departments of Strasbourg Hospital and the urological Clinic of Nantes, we collected 240 PCa samples from 237 patients. Human prostate cancer tissues were retrieved directly after surgery (radical prostatectomy, palliative TURP, surgical resection of node metastasis, pelvectomy) or biopsy (**Supplementary Table 1**). The use of patients’ tissues complied with a protocol approved by both the “Comité de Protection des Personnes Est IV” and the “Comité de Protection des Personnes Sud-Ouest et Outre-mer” (Approval numbers: *DC-2010-1193 and DC-2019-3565, respectively*). Patients enrolled in the study provided written informed consent allowing the use of discarded surgical samples for research purposes. In addition, relevant clinical information was recorded from the patients’ data including age, PSA levels, treatments, and treatment responses when available. Tumor regions within surgical specimens were identified by uro-pathologists. Once collected, prostate tissues were transported in an appropriate solution (Custodiol). Prostate tissues were implanted within 24 hr from collection.

### Animals

Four to five-week-old immuno-deficient mice including athymic Swiss-Nude (Crl : NU(Ico)-*Foxn1nu*), NMRI-Nude (Rj : NMRI-Foxn1^nu/nu^) or Shrn (NOD.Cg-Prkdc^scid^Hr^hr^/NCrHsd) male mice were purchased from Charles River Laboratories (L’Abresle, France), Envigo (Gannat, France) or Janvier Labs (Saint-Berthevin, France). Animals were handled under specific pathogen-free conditions. Their care and housing complied with guidelines set out in French animal welfare regulations referred as the European Community Council Directive 2010/63/UE. All tumorgraft studies were reviewed by CEE-35 and CEE-122 (ethical committees for the protection of animals used for scientific purposes) and approved by the French Ministry for National Education, Higher Education and Research under the numbers *APAFIS#2949-2015113017594629v5* and *APAFIS#14811-2018042316405732v6*. The animal facility was maintained under standardized conditions: artificial 12h light-dark cycles between 7:00 a.m. and 7 p.m., ambient temperature of 22 ± 2°C and relative humidity maintained at 55 ± 10%.

### Development of PDX models

The establishment of PDX models was carried out as previously described (21, 22). Briefly, different adult male immunodeficient mice strains were used for tissue implantation: Swiss-nude mice (G266, C901 and C1022) and Shrn mice (PCU-012 and PCU-018) for primo-implantations. At the time of grafting, mice were intact (G266, C901 and C1022) or had androgen supplementation (PCU-012 and PCU-018) (**Supplementary Table 2**). Grafts were implanted into the interscapular fat pad and monitored weekly for tumor growth for up to 9 months post-implantation for initial growth. When xenografted tumors reached ~1500 mm^3^, they were sequentially passaged into new mice under the same conditions and using the same protocol as the original implants. A PDX model was defined as established when stable growth over at least three passages and regrowth after a freeze-thaw cycle could be observed. At each mouse-to-mouse passage, representative samples were cryopreserved, snap-frozen in liquid nitrogen and/or FFPE processed.

### Immunohistochemistry

FFPE patient tumors and PDX blocks were sectioned and slides immunostained with the following antibodies: cytokeratin cocktail AE1AE3 (M3515, Agilent), androgen receptor (M3562, Agilent), hCD45 (IR751, Agilent), PSA (M0750, Agilent), ERG (AC-0105, Clinisciences), NKX3.1 (AC-0314, Clinisciences), PTEN (ab228466, Abcam), Ki67 (M7240, Agilent), P53 (GA616, Agilent), synaptophysin (IR660, Agilent) and chromogranin A (M0869, Agilent). After heat antigen retrieval as specified by provider, experiments were performed using Dako Omnis Instrument, EnVision FLEX, High pH kit for revelation (GV800, Agilent) for hCD45, cytokeratin cocktail AE1AE3, PSA, P53, synaptophysin, ERG and chromogranin A, or Leica Bond III Instrument for androgen receptor, Ki67 and NKX3.1 and on Autostainer 480S instrument for PTEN. The conditions are described in the **Supplementary Table 3**.

### Tissue processing for transcriptomic and genomic studies

Frozen samples were processed on ice. For DNA and RNA isolation, patient and PDX tumor fragments were processed using Qiagen Allprep DNA/RNA mini kit according to manufacturer’s instructions. Briefly, DNA and RNA were simultaneously extracted and purified. Nucleic acid yield and quality was assessed by NanoDrop spectophotometer ND8000 and RNA quality was further evaluated using Agilent 2100 Bioanalyzer.

### Short tandem repeat signature

The identity of each PDX was periodically authenticated by profiling STRs. Patient tumors and corresponding PDX DNA samples were subjected to STR using PowerPlex^®^ 16 HS System (Promega, ref DC2101) that amplifies 16 STR loci and the amelogenin gender-determining marker, according to manufacturer’s instructions. PDXs passed authenticity when >80% match in alleles was obtained. PCR products were separated by capillary electrophoresis on ABI prism 3500 and results were analyzed using GeneMapper software (v5).

### Whole exome sequencing

DNA extracted from tumorgraft tissues with adequate quality was subjected to WES by IntegraGen (France, Evry).

#### Sequence alignment and variant calling

Base calling was conducted using the Real-Time Analysis software sequence pipeline (2.7.7) from Illumina with default parameters. In order to remove contaminating mouse reads, raw reads were classified depending on their species of origin (graft or host) using the Xenome tool (23). Raw human reads were aligned on human hg38 genome using the Burrows-Wheeler Aligner (BWA) tool (24). Duplicated reads were removed using Sambamba (25). Variant calling of somatic single nucleotide variants (SNVs) and small insertions/deletions (indels) was performed using the Broad Institute’s GATK MuTect2 tool (2.0, –max_alt_alleles_in_normal_count=2; –max_alt_allele_in_normal_fraction=0.04) against a Panel of Normals (PON) comprising 107 normal samples sequenced by IntegraGen following the same protocol (26). Ensembl’ Variant Effect Predictor (27) (VEP, release 95) was used to annotate variants with respect to functional consequences (type of mutation and prediction of the functional impact on the protein by SIFT 5.2.2 and PolyPhen 2.2.2) and frequencies in public (dbSNP151, 1000 Genomes phase 3, gnomAD 17-02-28, COSMIC v86) and in-house databases.

#### Somatic variant analysis

To keep/detect only reliable somatic variants, the following post-filtering steps were applied:

-QSS score ≥ 20 (the average base quality of variant bases)
-coverage ≥10 in the tumor
-variant allele fraction in the tumor (VAF_T_)≥0.05 with ≥5 mutated reads
-gnomAD_Global_AF < 1e-5
-IntegraGen proprietary database AF < 0.01
-coding

We highlighted genes belonging to the list of 120 prostate cancer drivers defined by Armenia et al. (28).

#### Copy-number analysis using genotype data

Two complementary approaches were used to reconstruct the copy-number profiles of the tumors.

* Copy-number analysis using genotype data

We identified germline Single-Nucleotide Polymorphisms (SNPs) in each sample, and we calculated the coverage log-ratio (LRR) and B allele frequency (BAF) at each SNP site. Genomic profiles were divided into homogeneous segments by applying the circular binary segmentation algorithm, as implemented in the Bioconductor package *DNAcopy*, to both LRR and BAF values. We then used the Genome Alteration Print (GAP) method to determine the ploidy of each sample, the level of contamination with normal cells and the allele-specific copy number of each segment (29). Ploidy was estimated as the median copy-number across the genome. Chromosome aberrations were then defined using empirically determined thresholds as follows: gain, copy number > ploidy + 0.5; loss, copy number < ploidy – 0.5. We considered a segment to have undergone Loss of Heterozygosity (LOH) when the copy number of the minor allele was equal to 0.

* Copy-number analysis based on coverage

We calculated the coverage log ratio in each bait of the exon capture kit between the tumor and a panel of normal. Log-ratio profiles were then smoothed using the circular binary segmentation algorithm as implemented in the Bioconductor package *DNAcopy*. The most frequent smoothed value was the zero level of each sample. Segments with a smoothed log ratio above zero + 0.3 or below zero − 0.3 were considered to have gains and deletions, respectively. High-level amplification and homozygous deletion thresholds were defined as the mean +/- 5 s.d. of smoothed log ratios in normal regions, respectively. This approach does not provide absolute copy-number estimates but has a higher definition than the previous one as there are more exon capture baits than germline polymorphisms. It was used to characterize focal aberrations such as high-level amplifications and homozygous deletions.

Genomic analyses were performed with MERCURY™, an online biological interpretation tool for oncology. https://integragen.com/fr/bioinformatique/mercury

### RNA-Seq sequencing and analysis

Libraries were prepared with NEBNext^®^ Ultra™ II Directional RNA Library Prep Kit for Illumina protocol according to supplier recommendations.

Briefly, the key stages of this protocol are successively: the purification of PolyA containing mRNA molecules using poly-T oligo-attached magnetic beads from 100ng total RNA (with the Magnetic mRNA Isolation Kit from NEB); a fragmentation using divalent cations under elevated temperature to obtain approximately 300bp pieces; double strand cDNA synthesis, and finally Illumina adapter ligation and cDNA library amplification by PCR for sequencing. Sequencing was then carried out on Paired-end 100b reads of Illumina NovaSeq. Image analysis and base calling is performed using Illumina Real Time Analysis with default parameters.

### First analysis

Quality of reads was assessed for each sample using FastQC (V.0.11.4; http://www.bioinformatics.babraham.ac.uk/projects/fastqc/).

RNA-SeQC provided key measures of data quality. These metrics were shown within Reporting and included yield, alignment, and duplication rates, rRNA content, regions of alignment (exon, intron and intragenic). Alignment was performed by STAR (https://github.com/alexdobin/STAR).

The duplicate reads (*e.g.*, paired-end reads in which the insert DNA molecules have identical start and end locations in the Human genome) were removed using Sambamba tools (https://github.com/biod/sambamba).

### Second analysis

Variant calling for the identification of SNVs (Single Nucleotide Variations) and small insertions/deletions (up to 20bp) was performed *via* the Broad Institute’s GATK Haplotype Caller GVCF tool.

Ensembl’ VEP (Variant Effect Predictor, Release) program was used to process variants for further annotation. This tool annotates variants, determines the effect on relevant transcripts and proteins, and predicts the functional consequences of variants. This included considering data available in gnomAD, the 1000 Genomes Project and the Kaviar databases. Moreover, an in-house database enabled to filter out sequencing artefacts.

### Third analysis

Five bioinformatics algorithms for pathogenicity were used to predict the functional, molecular, and phenotypic consequences of coding and non-coding SNPs. This included DANN, FATHMM, MutationTaster, SIFT and Polyphen. The clinical and pathological significance was also added from the ClinVar database. Other information reported included quality score, homozygote/heterozygote status, count of variant allele reads, and presence of the variant in the COSMIC and OncoKB databases.

RegulomeDB was used to annotate SNPs in known and predicted regulatory elements in the intergenic regions.

### Fusion transcript analysis

To detect fusion-genes candidates in RNA-seq data, FusionCatcher (start with fastq files) and STAR-Fusion (start with alignment files) were used to achieve higher detection efficiency (30, 31). These were run using default configurations. In silico validation of a list of fusion transcript predictions was then performed using FusionInspector, a component of the Trinity Cancer Transcriptome Analysis Toolkit (CTAT) (32). FusionInspector assisted in fusion transcript discovery by performing a supervised analysis of fusion predictions, attempting to recover and re-score evidence for such predictions. Fusion-gene candidates were annotated according to several databases of known fusion genes found in healthy samples (known false positives) including the 1000 Genome Project, ChimerDB2, GTEx and cancer databases such as COSMIC, 18Cancers.

### Expression

Counting of reads per gene was performed using STAR with –quantMode GeneCounts option. Next, raw count was normalized using the Transcripts Per Kilobase Million (TPM) method.

Analyses were performed with Galiléo™, a cloud-based app for dynamic exploration of RNA-Seq expression data. https://integragen.com/fr/bioinformatique/galileo

### *In vivo* efficacy studies

For preclinical *in vivo* drug testing, tumor fragments were implanted into the interscapular fat pad of NMRI nude immunodeficient mice strain as described above. When tumors reached a volume comprised between 65 and 270 mm^3^, mice were randomly assigned to the vehicle or treatment groups (n=6-8 per group). Mice were then treated with 20 mg/kg docetaxel (1 dose every 3 weeks by intraperitoneal route, MedChemExpress, HY-B0011), 12 mg/kg leuprorelin (1 dose/week by subcutaneous injection, MedChemExpress HY-13665), 60 mg/kg enzalutamide (5 doses/week by oral gavage, MedChemExpress HY-70002), 200 mg/kg abiraterone (daily by oral gavage, MedChemExpress HY-70013) and 75 mg/kg olaparib (daily by oral gavage, MedChemExpress HY-10162). Olaparib was also administered in combination with abiraterone and enzalutamide at the same doses and schedule. Drugs were prepared in different solution; in 20% of a mix (50% Tween80 + 50% Ethanol), 80% NaCl 0.9% for docetaxel, in NaCl 0.9% for leuprorelin, in 10% DMSO + 40% PEG300 + 50% (0,5% Tween80) in NaCl 0.9% for enzalutamide, and in 10% DMSO + 90% (10% HP-β-CD) in PBS 1x for abiraterone and olaparib. Mice were treated until one animal reached the maximum ethical limit tumor volume of 1500 mm^3^ and/or a body weight loss > 20% for 3 consecutive measurements compared to the first day of treatment, in this case the entire group was removed from the study. Tumor volume was measured twice a week with a caliper and calculated as: TV (mm^3^) = [length (mm) x width (mm) ^2^]*π/6, where the length and width are the longest and shortest diameters of the tumor, respectively. For evaluation of therapeutic response, tumor growth was calculated as ΔT/ΔC in percentage where ΔT and ΔC are tumor volume changes relative to initial mean tumor volume for treated group (T) and control group (C), respectively, at a specific day. Response to treatment was also classified using the modified Response Evaluation Criteria in Solid Tumors (mRECIST) using the percentage of tumor volume change at the last day of treatment compared with the tumor volume at day 0 and classified as follows: complete response, BestResponse < −95% and BestAvgResponse < −40%; partial response, BestResponse < −50% and BestAvgResponse < −20%; stable disease, BestResponse < 35% and BestAvgResponse < 30%; progressive disease, not otherwise categorized (33). Health status and body weight for all mice were recorded twice weekly to control any adverse effects.

### Statistical analysis

Comparisons between PDX treatment responses were presented as mean ± SEM. ^ns^, P>0.05; *, P<0.05; **, P<0.01; ***, P<0.001; ****, P<0.0001 comparing treated to control groups using a two-way ANOVA followed by a Dunnet’s multiple comparisons post-test.

## Results

### Establishment of prostate PDXs

The aim of the present work was to generate PCa PDX models for preclinical applications. To this end, we worked in close collaboration with the Urology department of Strasbourg Hospital and the urological Clinic of Nantes to collect specimens from patients who had undergone surgery or, in a few cases, biopsy. Between 2010 and 2019, we processed 240 PCa samples from 237 patients. Collected samples were obtained from therapeutic or diagnostic procedures: 205 prostatectomies (85%), 31 transurethral resections of the prostate (TURP) (13%), 1 pelvectomy and 1 directly after biopsy (**Supplementary Table 1**). All men with a presumed diagnosis of PCa were eligible. The median patient age was 64 years (range 43 - 89 years). All the implanted tumors were obtained from localized or regionally advanced diseases, namely 238 primary tumors and 2 samples that were derived from regional lymph node metastasis (**Supplementary Table 1**). PDX models were developed from patients across the disease progression, *i.e.*, treatment-naïve to castrate resistant disease.

Viable tumor tissues were xenografted subcutaneously into various strains of immune-deficient mice (for details see materials and methods). For each graft, 2 to 5 mice were used depending on material availability. Eighteen primary implants gave rise to a first tumor growth in mouse: 67% originated from prostatectomies (11 RP and 1 pelvectomy out of 18), 28% from specimens harvested following TURP (5 out of 18) and 5% from lymph node metastasis (1 out of 18). We obtained a low take rate probably due to the variability of the amount of viable tissue submitted for PDX development, mouse strain used and engraftment site. It took between 2.5 and 7.5 months to observe the first growth in mouse irrespectively of tumor stage. Nine tumors that grew did not survive after passage 1. The other half (9 tumors) were successfully propagated beyond three passages. Unfortunately, 3 models did not grow after freezing/thawing despite attempts in several strains of mice and 1 model was contaminated by lymphoma.

In total, we established 5 sequentially transplantable PDXs as working models. PCU-012 and PCU-018 PDX models were derived from primary local tumor samples from treatment-naïve patients. C901 PDX model was obtained from TURP. At the time of surgery, the corresponding patient was responsive to androgen-deprivation therapy (ADT). Cancer progressed and the patient underwent an additional palliative TURP eight months later. TURP chips were obtained and successfully implanted to generate the C1022 PDX model, which displays a neuroendocrine phenotype. PCa samples for the establishment of G266 were obtained at pelvectomy and correlated with a recurrence. **Supplementary Table 4** summarizes the whole clinical annotated data from patients from whom PDXs were derived.

### Histological and genomic characterizations of PDXs

#### H&E

**Figure 1:**
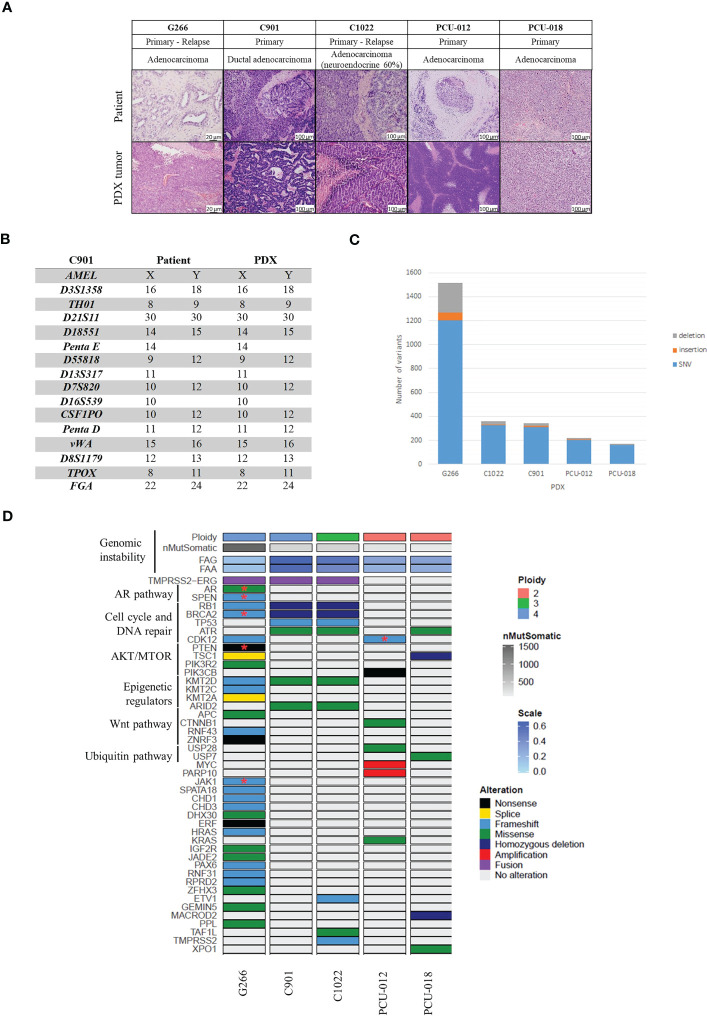
Histological and genomic characteristics of patient’s tumors and paired derived patient-derived xenografts (PDXs). **(A)** Comparative analysis between patient tumor and corresponding PDX model assessed by hematoxylin and eosin **(H&E)** staining and demonstrating feature preservation. Scale bar corresponds to 100 μm except for G266 (20 µm). H&E slides were reviewed by a board-certified pathologists and representative pictures are shown. **(B)** Short tandem repeat signature of a patient specimen and PDX tumor, an example of the C901 case. **(C)** Number of variants identified. **(D)** Somatic genomic landscape of 5 prostate PDXs analyzed using a whole exome sequencing approach.​ FAA (Fraction of Aberrant chromosome Arms); FAG (Fraction of Aberrant Genome); SNV: single nucleotide variation; Stars indicate two mutations in the same gene​.

A histopathological comparative analysis of the H&E-stained slides of patient tumors and PDXs was performed. In 4/5 cases, the morphological features of the PDX replicated closely the human primary tumors. PCU-012 primary tumor and PDX model were Gleason 5 + 4 with solid pattern and scattered glandular lumens. PCU-018 PDX model had the same pattern of pleomorphic giant cell adenocarcinoma as observed for the primary tumor (Gleason 5 + 5). C901 PDX displayed the same cribriform and complex papillary pattern (equivalent Gleason 4 + 4) as for the primary tumor classified as ductal adenocarcinoma (Gleason 5 + 4). C1022 human tumor showed two distinct components, an adenocarcinoma with the same cribriform/papillary features described for C901, and a neuroendocrine small cell carcinoma with sheets of basophilic cells and extensive necrosis; the C1022 PDX mimicked only the neuroendocrine carcinoma component. G266 patient tumor and PDX were both graded Gleason 5 + 5 (**Figure 1A**).

#### STR

Using Short Tandem Repeat (STR) profiling, we confirmed the concordant genetic identity between patient tumors and derived PDXs (**Supplementary Figure 1**), with 100% of conserved STR for 4 models, an example of C901 STR profile is shown in **Figure 1B**. PDX G266 appeared to have minor alleles differences between the patient sample and the derived PDX with a homology rate superior to 80%. Discrepancies were due to the ploidy of the primary tumor since the Copy Number Alterations (CNA) revealed a tetraploid genome (**Supplementary Figure 2A**).

#### Genomic alterations

We investigated genomic alterations for cancer-related genes in all 5 PDXs using a whole exome sequencing (WES) assay, which enabled the detection of mutations (**Supplementary Table 5**), CNA (**Supplementary Tables 6**, **7**), tumor mutational burden (TMB), and MSI status (**Figure 1** and **Supplementary Figure 2**).

We initially considered 97 cancer driver genes and added 23 genes with unknown but recurrent significance (28, 34, 35). The frequency of alterations for each gene was compared to data obtained by Armenia et al. (28). As expected, the main altered pathways were the AR, the cell cycle and DNA repair, *AKT/mTOR*, epigenetic regulators, Wnt and the ubiquitin pathways.

PDX G266 harbored the highest mutational burden (30,99 variant/Mb) (**Figure 1C** and **Supplementary Figure 2B**) followed by PDXs C901 (8,46 variant/Mb), C1022 (7,64 variant/Mb), PCU-012 (5,99 variant/Mb), and PCU-018 (3,6 variant/Mb), respectively. G266 displayed a microsatellite instability profile with a homozygous deletion of *MSH2* and a loss of *MSH6*. PDXs G266, C901 and C1022 shared a mutation profile representative of those observed in prostate metastatic tumors such as *TMPRSS2-ERG* fusion, *TMPRSS2*, *RB1*, *BRCA2*, and *KMT2D* mutations. For C1022, TMPRSS2-ERG fusion was not detected first and analyzed by Mercury™, probably because it was present in an inferior percentage of cells in C1022 compared to C901 and filtered as not supported by a sufficient number of reads. Based on WES data, this TMPRSS2-ERG fusion was deduced due to the presence of a small deletion on chromosome 21, where the terminals coincided specifically with those genes (**Supplementary Figure 3**). The deletion appeared more clearly within the genome of PDX C901 but the same breakpoints were found on the logR ratio profile of PDX C1022. G266 and C1022 profiles were consistent with the patients’ tumor history since they were relapses from a primary tumor and were more aggressive than the other patients’ tumors. C901 and C1022 had a homologous deletion of both *BRCA2* and *RB1*, and G266 harbored a deletion leading to a frameshift. Together with other mutations implicated in DNA homologous recombination repair genes, these three PDX models had an eligible profile for pharmacology studies with PARP inhibitors. G266 was the only one to demonstrate *AR* missense activating mutations (p.(Thr878Ala)) and (p.(Trp742Cys)) present in castration-resistant disease but also a *PIK3R2* missense (p.(Gly373Arg)) and *PTEN* nonsense mutations (p.(Arg233Ter)) stop gained).

PCU-012 had a profile compatible with a genomic instability associated with a CDK12 inactivation, copy number amplifications dispersed across the genome, a copy number aberration on chromosome 8q with focal amplification of *MYC* and *PARP10* and a high number of gene fusions compared to other PDXs. Two alterations were found in the *CDK12* gene, a frameshift in exon 6 and a pathogenic missense mutation in exon 10 (p.(Cys952Arg)) in the kinase domain of the protein (**Supplementary Table 5**). The associated copy number was 2.52 with a high expression level of *CDK12* transcript. This model also presented a deletion in *BRCA2* (p.(Cys1200Ter)) leading to a frameshift and mutations in two hot spot genes, *CTNNB1* (p.(Asp32Tyr)) and *KDR* (p.(Cys482Arg)). No *ETS* fusion, nor *PTEN*, *ATM* or *SPOP* mutations were detected which was consistent with the CDK12 phenotype.

PCU-018 had a diploid profile with an important number of deletions (383 deleted cancer genes) spread widely all over the genome with homozygous deletions of the tumor suppressor *TSC1* and *MACROD2*, a hydrolase, which removes mono-ADP-ribosylation and which is implicated in chromosome instability in colorectal cancer (36). Few gains (2 amplifications and 77 gains) and 9 fusions of interest were also found. Three portions of copy neutral loss of heterozygosity (CN-LOH) in chromosomes 1 (77 mb), 11 (11 mb) and 17 (56 mb) were found, respectively. Missense mutations were identified in 3 cancer gene drivers: *ATR* (p.(His4Tyr)) and Rad3-related protein, implicated in replication stress response (37); *USP7* (p.(Arg634Asp)) for ubiquitin specific protease 7, member of the deubiquitinating enzyme family (38); and *XPO1* (p.(Ser1031Thr)) a nuclear export protein implicated in cellular homeostasis (**Supplementary Table 5**) (39).

Taken together, these data highlighted the genetic heterogeneity between our PDX models with common mutations in those from advanced PCa compared to localized ones. They were consistent with genomic aberrations previously described in literature regarding their phenotype and grade.

#### CRPC-NE and HSPC tumors appear clonal in origin with clonal ancestry

C901 and C1022 PDX models were derived from tumor samples from the same patient at two different time points. The patient was diagnosed with an adenocarcinoma and treated with adjuvant ADT before surgery. He had a transurethral resection of the prostate TURP for a clinical localized Gleason 8 (4 + 4) prostate adenocarcinoma and was treated with ADT. The cancer relapsed eight months later and an adenocarcinoma with 60% of neuroendocrine component was removed (**Supplementary Table 4**). C901 was tetraploid and C1022 triploid. The mutations of the two PDXs derived from these surgeries were analyzed and compared. They shared 245 mutations. 112 and 129 were specific to C901 and C1022, respectively (**Supplementary Figure 2C**). Six non-synonymous mutations were identified in cancer driver genes. Mutations of *TP53* (p.(Arg335ValfsTer10) frameshift), *ARID2* (p.(Glu44Asp)) and *KMT2D* (p.(Lys5091Arg)) were present in both PDXs as well as the TMPRSS2-ERG fusion, although less were detected in C1022. Two homozygous deletions on chromosome 13 implicating tumor suppressor genes *BRCA2* and *RB1* were also observed (**Figure 1D** and **Supplementary Table 5**). *TMPRSS2* (p.(Ser234GlufsTer16)) frameshift, *ETV1* (CN-LOH) and *TAF1L* (*p.*(Val495Glu)) mutations were only present in C1022. The *TMPRSS2* mutation was a translocation between exon 7 and intron 2 of the enzyme β-carotene oxygenase 1, *BCO1*, a survival prognostic gene (40).

Taken together, these results suggested that these two PDXs models had a common precursor with common mutations but had then evolved separately, under ADT (**Supplementary Figure 4**) (41).

### PDXs recapitulate the molecular subtypes of prostate cancer

#### Immunohistochemical phenotypes

**Figure 2:**
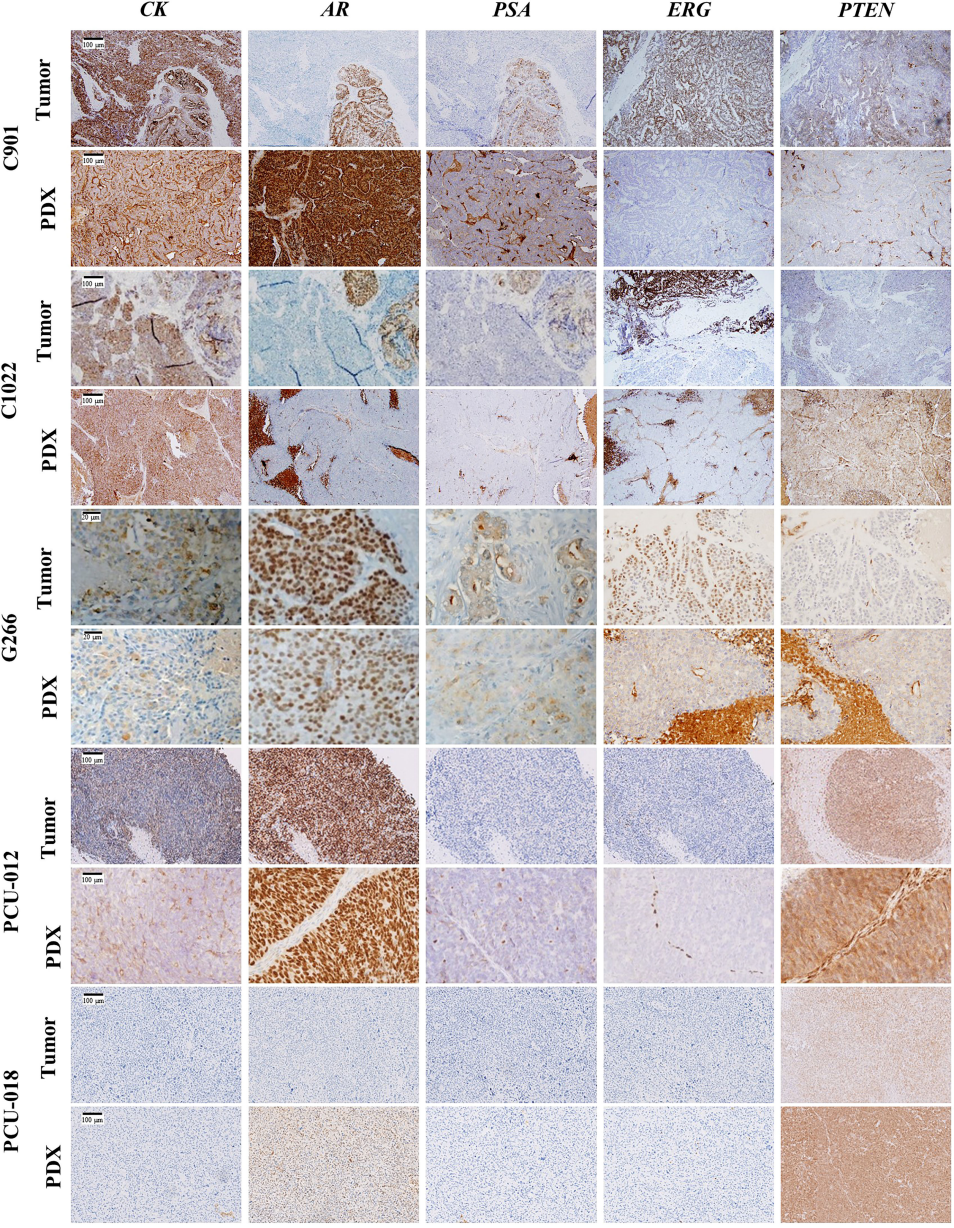
Immunohistochemical characterization of the 5 PCa PDX models. Representative immunochemical staining for CK (cytokeratin), AR (androgen receptor), PSA (prostate specific antigen), ERG, and PTEN in patient tumor and corresponding PDX model. Scale bar corresponds to 100 µm unless specified.

A nuclear ERG expression was observed for the C901 primary tumor and PDX, as well as in the adenocarcinoma component of human tumor C1022, consistent with the *TMPRSS2-ERG* fusion detected in C901 and C1022 PDX. Of note, ERG was not expressed in the C1022 human tumor and PDX neuroendocrine carcinoma, however a strong expression of synaptophysin and chromogranin was measured confirming the NE phenotype (**Supplementary Figure 5**). A loss of PTEN expression was observed for the G266 primary tumor and corresponding PDX in agreement with the non-sense *PTEN* mutation detected in the PDX. The other primary tumor/PDX pairs showed a conserved expression of PTEN. Among the two PDX derived from hormonal treatment naïve tumors, the androgen receptor expression ranged between low (PCU-018) to high (PCU-12). The expression was also high for the G266 and C901 PDX derived from a tumor collected in a hormone treated patient. It was absent within the C1022 neuroendocrine carcinoma (**Figure 2**). NKX3.1 expression was detected in all tested PDX, to a lower extend for PDX C1022 consistent with its neuroendocrine profile (**Supplementary Figure 5**). PDXs were stained for hCD45, a nonspecific lymphocyte marker, to evaluate human lymphoma presence (**Supplementary Figure 5**). No expression was seen for 4/5 models, a low expression associated with background noise was seen for the C901 PDX model. The stained areas are free of cells or contain red blood cells.

#### Hierarchical clustering

**Figure 3:**
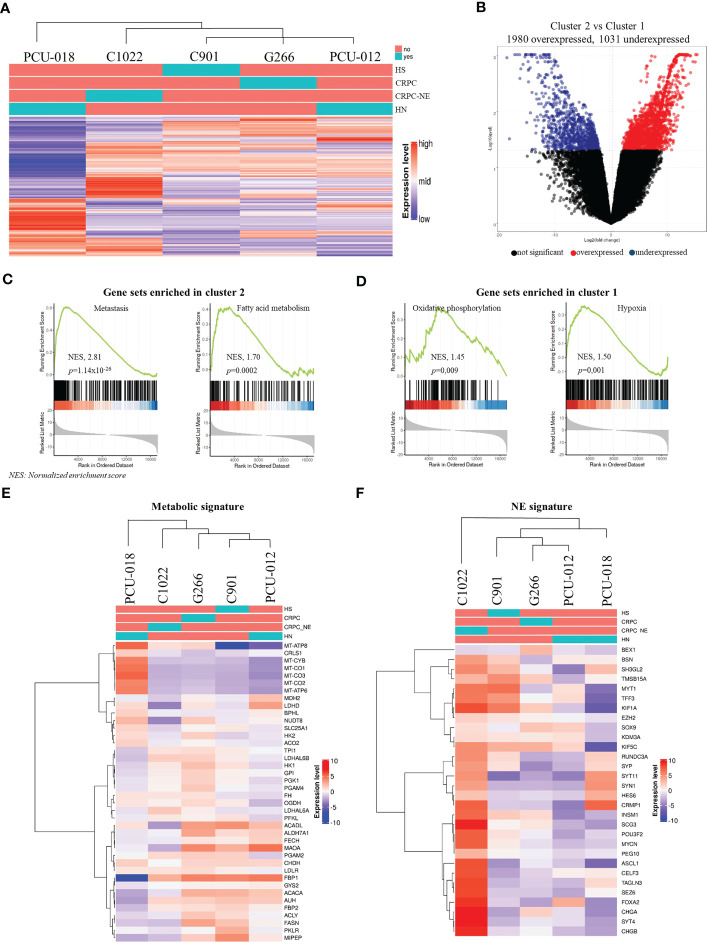
Analysis of gene expression by RNA sequencing of prostate cancer PDXs. **(A)** Unbiased hierarchical clustering with a color code panel depicting treatment status (naïve or treated), castrate resistant and neuroendocrine status (CRPC and CRPC-NE) based on genes with the most variant expression (n=1000). **(B)** Volcano plot representation showing top up and down-regulated genes in tumor with metastatic molecular features (Cluster 2) vs. localized tumors (Cluster 1). **(C, D)** Gene set enrichment analysis (GSEA) analysis of RMA normalized gene expression. Biological processes are significantly different between Cluster 2 and Cluster 1. **(E, F)** Heatmap of gene expression signature for the selected gene signature: metabolic gene signature and neuroendocrine gene signature.​ (HS, hormone-sensitive; CRPC, castrate resistant prostate cancer; CRPC-NE, castrate resistant prostate cancer neuroendocrine; HN, hormone-naïve; NES, normalized enrichment score).

We then sought to determine whether our PDX models could be segregated by hormone-sensitive or castrate-resistant tumor phenotype using an unsupervised clustering analysis based on the topmost 1 000 variant genes after RNASeq (**Figure 3A**). No segregation regarding treatment sensitivity was found but rather a stratification separating PDXs derived from primary tumors from PDX tumors derived from recurrent or metastatic tumors. Derived from a primary localized tumor, PCU-018 was clustered alone. PDXs G266, C1022, C901 and PCU-012 belonged to the same cluster but C1022 appeared in a different subcluster as it was from a CRPC-neuroendocrine tumor. Interestingly, although derived from a primary localized tumor, PCU-012 was also clustered with PDXs derived from recurrent and metastatic tumors. This was consistent with the patient’s outcome since metastases developed shortly after surgery. This supports the accuracy of a gene signature in localized prostate cancer that can predict whether the cancer is likely to spread, or metastasize, early in the course of the disease.

#### GSEA (Gene Set Enrichment Analysis)

Among all 3 011 differential mRNAs, 1 980 genes were over-expressed, and 1 031 genes were under-expressed in cluster 2 (C1022, C901, G266 and PCU-012) compared to cluster 1 (PCU-018) (**Figure 3B**, **Supplementary Table 8**). To explore this result further, we performed a GSEA of the MSigDB collections between cluster 1 and cluster 2. GSEA revealed the enrichment of several expected pathways for cluster 2 such as for the gene associated to a metastatic phenotype (NES, 2.81; p=1.14 x 10^-26^). Notably, cluster 2 samples showed overexpression of genes implicated in fatty acid/glycolysis metabolism, whereas cluster 1 samples overexpressed genes involved in oxidative phosphorylation (NES, 1.45; p=0.009) consistent with the metabolic reprogramming occurring across PCa progression (**Figures 3C, D**). Additionally, a pathway analysis between the two clusters highlighted the top up and down pathways. Cluster 2, associated with a metastatic phenotype, showed upregulation of androgen/estrogen pathways whereas cluster 1 (localized tumor) highlighted upregulation of inflammation response and IL6-JAK-STAT3, IL2-STAT5 pathways.

#### Metabolic reprogramming in prostate cancer

We subsequently focused our analysis on metabolism since recent studies have revealed new insights into specific PCa metabolic reprogramming vulnerabilities that can be targeted (42–45). The metabolism gene signature of the 5 PDX models recapitulating some genes involved in oxidative phosphorylation/mitochondrial DNA, in glycolysis and lipid metabolism showed the same clustering as for the topmost 1 000 variant genes (**Figures 3A–E**). PCU-018 highly expressed oxidative phosphorylation (OXPHOS) and mitochondrial genes such as *ACO2*, *FH* and *OGDH* involved in tricarboxylic acid (TCA) cycle and *MT-CO1*, *MT-CO2*, *MT-CO3* coding for mitochondrial cytochrome c oxidase and *MT-ATP6*, *MT-ATP8* coding for ATP synthase (**Figure 3E**). On the other hand, late stage PDX tumors overexpressed a clear subset of genes implicated in glycolysis, fatty acid (FA) oxidation and lactate production such as *FBP*, *FASN* or *LDH* genes. Several studies have shown that TCA cycle dysregulation through OXPHOS inhibition leads to an increased expression of *ACACA* and *FASN* genes, suggesting an enhanced FA synthesis and PCa progression (46, 47). Interestingly, the *MAOA* gene which is shown as responsible for mitochondrial dysfunction and glycolysis promotion in gastric cancer was over expressed in PCU-012, G266 and C901 (48). Overall, these data reflected the metabolic changes that occur in PCa, first from aerobic/anaerobic glycolysis in normal prostate cells to oxidative phosphorylation of cancer cells, and then in metastasis, glycolytic activities and increased oxidation of fatty acids (49). This opened up the possibility to target OXPHOS and mitochondrial activities in PCa to prevent the metabolic switch required for PCa progression (50, 51) and to defuel advanced PCa with metabolic inhibitors (44, 52).

It could be noted that C1022 CRPC-NE PDX also appeared in a different subcluster with an intermediate metabolic state. Neuroendocrine PCa AR-null phenotype presents an unmet clinical challenge and requires further investigation of its metabolic regulation which is still not fully understood. Choi et al., reported enhanced glycolysis associated with lactic acid production in NEPC and proposed to target MCT4 (53); while other groups reported the significance of fatty acid (FA) metabolism, glycolysis or the importance of the carnitine palmitoyl transferase I (CPT1) (54–57). Interestingly, our neuroendocrine PDX model did not express *MCT4*; no over expression of *CPT1* or specific metabolic pathway was noticed, suggesting heterogeneity of neuroendocrine prostate tumors already described (58–60).

#### NE signature

To validate the neuroendocrine profile of C1022, we evaluated PDX patterns using previously defined NE signatures (61, 62). As expected, the NE subtype PDX was markedly different from non-NE PDX (**Figure 3F**) and had a pattern of expression in accordance with its neuroendocrine profile, with an absence of *AR* expression, *RB1* loss, low *TP53* expression, *MYCN* overexpression as well as upregulation of *POUF2R3*, *SOX2* and *PEG10* and *ERG* rearrangement (**Supplementary Figure 6B**). Additionally, the neuroendocrine chromogranin A and synaptophysin markers were diffusely expressed in C1022 primary tumor and PDX, supporting the diagnosis of poorly differentiated neuroendocrine carcinoma (**Supplementary Figure 5**).

C1022 PDX also overexpressed epigenetic factors such as *SRRM4* and *EZH2* and showed a downregulation of *REST*, all implicated in tissue plasticity (**Supplementary Figure 6B**) (63). Interestingly, PDX C901 revealed expression of some NE genes, which could have predicted the fate of this tumor.

#### Predictive evolution of the tumor

AR is known to be a key regulator that orchestrates metabolic reprogramming depending on the stage of disease progression (64). Indeed, PDX’s metabolism profiles can be linked to AR status with a drop of oxidative/fatty acid metabolism associated to loss of AR expression for C1022 (**Supplementary Figure 6C**). These results highlighted the link between metabolic pathways, AR signaling and the stage of the tumor, as well as the importance of identifying the metabolic weaknesses of PCa.

Overall, these molecular signatures highlighted the importance of this analysis as a particularly informative resource for predicting tumor progression and tailoring patient treatment accordingly, as it could have been done for tumor C901 which already expressed NE signature, or for PCU-012, which showed a metastasis signature even though it was a local tumor.

### Sensitivity of patient-derived xenografts to standard of care treatment

**Figure 4:**
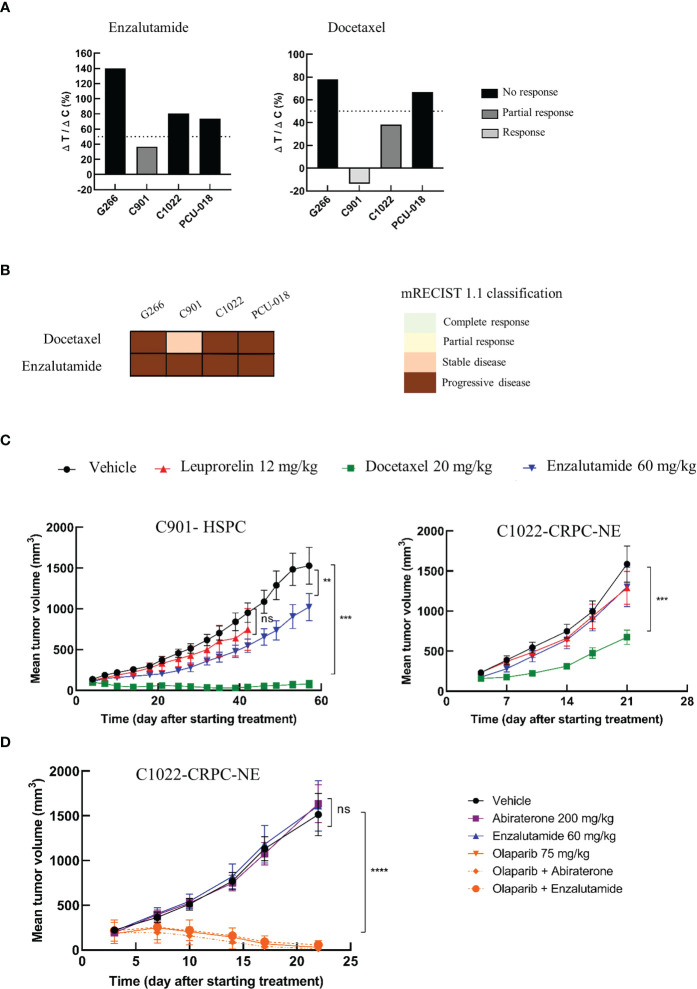
Preclinical testing of a panel of therapies from chemotherapy to targeted therapy. Mice (n=6-8 per group) with established PDXs (65 mm^3^ - 270 mm^3^) were treated with various drugs or control vehicle. Tumors were measured at the indicated time points. **(A)** PDX response to docetaxel and enzalutamide according to tumor growth inhibition (TGI) presented as the ΔT/ΔC ratio and calculated as the ratio of the mean tumor volume for the treated vs. control group, with a response when ΔT/ΔC < 0%, a partial response when ΔT/ΔC 0-50% and no response when ΔT/ΔC >50%. **(B)** PDX response to docetaxel and enzalutamide treatment according to modified mRECIST 1.1 classification. **(C)** Growth response curves of C901 (androgen-dependent) and C1022 (castrate-resistant) PDX models generated from the same patient to docetaxel, leuprorelin and enzalutamide administrations. **(D)** Sensitivity of the castrate resistant neuroendocrine PDX (C1022) to androgen receptor inhibitors, to olaparib, a PARP inhibitor, given alone or in combination. Data are presented as mean ± SEM. ^ns^P>0.05; **P<0.01; ***P<0.001; ****P<0.0001 comparing treated to control groups using a two-way ANOVA followed by a Dunnet’s multiple comparisons post-test​.

As described above, the PDX panel presented tumors with diverse clinical and genomic characteristics across the course of PCa disease, allowing to evaluate drug efficacy on either treatment-naïve tumors, hormone-sensitive tumors, or castrate-resistant tumors. We investigated the sensitivity to docetaxel, a standard of care chemotherapy, and enzalutamide, a new generation androgen pathway inhibitor in our established PDX cohort (**Figure 4A**, **Supplementary Figure 7**). The response to each treatment reflected the heterogeneous clinical and genomic characteristics of the tumors. As expected, both G266 and C1022 CRPC PDX models were resistant to enzalutamide while the hormone-sensitive C901 PDX model was sensitive and exhibited a partial response. C901 and C1022 PDX models, derived from the same patient, were sensitive to docetaxel with a clear response for C901 and a partial response for C1022, while G266 was resistant (**Figure 4A**). Treatment responses of these two models reflected the hormone-resistance acquisition of the patient over time. Interestingly, PCU-018 from a localized and treatment-naïve tumor, was resistant to docetaxel and enzalutamide (**Figure 4A**).

In a clinical situation, the Response Evaluation Criteria in Solid Tumor (RECIST) are prevalently used to assess treatment responsiveness. Correspondingly, modified RECIST (mRECIST) was suggested to evaluate the treatment response in PDX model (33, 65). The mRECIST is estimated using the percentage of tumor volume change at the last day of treatment compared with tumor volume at day 0. We considered “responders” PDXs that showed a complete response (CR), partial response (PR) or stable disease (SD) and “non-responders” those with a progressive disease (PD) status. C901 was responsive to docetaxel treatment with a negative tumor volume change indicating shrinkage of the tumor after treatment, even though a tiny ball remained palpable; whereas using the modified RECIST classification, C901 was classed as a stable disease (SD) and not a responsive tumor (**Figure 4B**). The tumor response associated with C1022 was classed as a progressive disease (PD).

We then focused on C901 and C1022 PDX models which are interesting as they can provide a relevant preclinical tool to identify resistance mechanisms and to develop new therapeutic strategies. Both models were treated with androgen deprivation therapies (ADT), leuprorelin and enzalutamide, and with the chemotherapeutic docetaxel for at least 21 days. The hormone-sensitive model, C901, displayed relatively slow growth compared to the neuroendocrine castrate-resistant model C1022 as it took 60 days to reach tumor ethical size in the C901 vehicle group compared to 21 days for C1022 (**Figure 4C**). C901 tumor growth was significantly delayed when treated with enzalutamide while C1022 was a non-responder, correlating with their respective characteristics. Both models were responsive to docetaxel treatment, but an acquired resistance appeared for C1022. None of these two PDX responded to leuprorelin treatment which was used to treat the patient before and after the first surgery, the tumor of whom became resistant at the time of the second surgery, eight months later. Collectively, these analyses shed light on the occurrence of resistance in the same patient and the possibility of studying this through corresponding PDXs. Furthermore, the characteristics of PDX C1022 were consistent with the differentiation of neuroendocrine tumors, which were characterized by a highly proliferative profile, lack of responsiveness to hormonal therapies, and poor prognosis (66, 67).

### Inhibition of PARP significantly improves antitumoral response of CRPC-NE PDX compared to ADT

PARP inhibitors have recently been approved for patients presenting defects in homologous recombination repair (68, 69). Since the neuroendocrine castrate resistant C1022 model was unresponsive to all tested drugs and had a homologous deletion of *BRCA2*, we assessed the antitumor activity of the PARP inhibitor, olaparib, on this model. Given that previous works have demonstrated the improved efficacy of olaparib compared to androgenic pathway inhibitors (70) and given that clinical trials are now focusing on combining PARP inhibitors with other treatments such as androgenic pathway inhibitors (71), we decided to evaluate the efficacy of olaparib also in combination with either enzalutamide or abiraterone. As previously observed (**Figure 4C**), this rapidly growing PDX reached maximum ethical tumor volume between days 21 and 22. The response to androgenic pathway inhibitors was also consistent with our previous results; C1022 PDX was unresponsive to enzalutamide and presented no response to the other inhibitor abiraterone (**Figure 4D**). Tumor growth was drastically reduced upon treatment with olaparib. In some mice, no tumors were even detectable at the end of the study. Of note, no adverse effects were noticed in any of the treated groups (data not shown).

Taken together, these data were consistent with the patient’s tumor responses and paved the way to new therapeutic strategies for patients with CRPC-NE.

## Discussion

Preclinical relevant models for drug development are still very much needed. Genetic and molecular characterizations of PDX models have confirmed their utility as avatars for testing new therapies and combinations as they maintain a high degree of molecular fidelity to the original patient’s tumor (33, 72–74). PCa PDX models have been shown to capture the biological and molecular heterogeneity of the patient’s tumor, preserved histopathology features as well as genome architecture and global gene expression (75). However, their development remains challenging as their take rate is amongst the lowest, they proliferate very slowly and only high grade PCa with Gleason scores above 8 culminate in research ready to use preclinical models (15, 76).

We report here, like others, that our collection of 5 PDXs derived from PCa preserved the histological and molecular properties of patients’ tumors (15, 77).

From weakly to highly rearranged genomes, these PDX models reflect the heterogeneity of genomic variations in PCa encompassing different patterns of alterations. We described potential actionable genetic drivers such as *MYCN* and *PARP10* (78) that are amplified in the PCU-012 model. These mutations were sufficient to induce a CDK12 phenotype as described by Wu et al. (79).

We also described a drug response effect using new therapies such as the PARP inhibitor olaparib with our CRPC-NE PDX model that, used as a monotherapy, could reduce tumor growth significantly. Combination therapy did not improve efficacy, probably due to the high dose of olaparib, which, already greatly reduced tumor volume alone and because of the neuroendocrine nature of the C1022 tumor given that abiraterone and enzalutamide are efficient on AR+ tumors. In order to optimize treatment and evaluate a potential additive or synergistic effect for combination treatments, it would be interesting to explore the effect of olaparib at graded doses on *BRCA2* and AR+ mutated PDX models. Overall, these data confirmed the efficacy of PARP inhibition on *BRCA2*-mutated tumors. This opens the field for targeting other Homology Directed Repairs (HDRs) in PCa, including the aggressive neuroendocrine subset that presents very few treatment options. Beyond PDXs with *BRCA2* mutations, those PDXs with deficiencies in other DNA-damage repair-associated genes *(e.g.*, *PALB2*) should be eligible to treatments with PARP inhibitors since they may benefit from PARP inhibition, as suggested by Abida et al. (80). With its MSI-H profile, G266 should be a good model for testing immune checkpoint inhibitors (81). Our models could thus be useful for evaluating such new potential therapies alone or in combination.

We identified two clusters according to their hierarchical expression pattern characterization: a cluster with PDXs derived from primary localized tumors and a cluster derived from advanced tumors. It was interesting to observe that these two clusters could be further characterized by their metastatic potential, as highlighted by the expression levels of both the genes involved in metastasis processes and EMT and FA genes known to be key instruments of tumor dissemination. This information is crucial in the choice of the patient’s further treatment, as demonstrated by our PDXs PCU-012 and C901: both of which were derived from local primary tumors, but which already harboured the molecular signatures of epithelial to mesenchymal transition and FA metabolism. It has been previously published that repeated exposure to anticancer therapies may select carcinoma cells with partial mesenchymal phenotype coincident with the emergence of drug resistance (82, 83). The C901 PDX model, although hormone-sensitive, already mirrored drug resistance with its intrinsic tumor heterogeneity. This was supported by the stable disease (SD) state measured with mRECIST classification, where a residual tumor was still measurable after treatment with enzalutamide. It would be of interest to test different therapies based on the expression pattern analysed combining anti-MYC and PARP inhibitors with metabolic inhibitors for the PDX PCU-012 with a CDK12 phenotype.

As described previously, the remarkable plasticity in lineage identity of PCa cells might explain the development of ADT resistance and the emergence of CRPC and CRPC-NE phenotypes (63). This could be observed thanks to a further analysis of epigenetic drivers responsible for cellular plasticity and/or neuroendocrine differentiation (41, 84–86). High expression levels of those biomarkers of plasticity were measured in PDX C1022, probably promoting lineage, resistance to androgen-targeting therapies and confirming its NE phenotype. PDXs C901 and C1022 illustrate the selective pressure under ADT from the primary tumor that was hormone sensitive until the CRPC-NE phenotype.

Finally, we analysed the expression of biomarkers of the metabolism. The dysregulation of metabolism in cancer cells was first described a hundred years ago and is now being reconsidered taking into consideration non-cancer cells from the tumor microenvironment (87). The TCA cycle is truncated in normal epithelial cells from the prostate and low rate of oxidative phosphorylation is observed (OXPHOS) (88). In PCa with genetic alterations such as PTEN loss, P53 loss and MYC overexpression, the TCA cycle is reactivated for energy production and *de novo* lipid synthesis. The Warburg effect or aerobic glycolysis is observed in the metastatic stages of the disease. In accordance with the literature, our clustering highlights the metabolic shift in PCa cells with metabolic vulnerabilities that could be novel targets in PCa. As an example, the PCU-018 PDX model is an interesting tool to test small-molecule metabolic inhibitors of the mitochondrial oxidative phosphorylation (89) alone or in combination with other therapies. G266, C901 and PCU-012, according to their profile, could be used to test ACLY and FASN inhibitors (90, 91). With its neuroendocrine phenotype, C1022 appeared to have a different metabolic profile which did not correspond to those already described, opening up other possibilities for new targets (92).

The different molecular signatures identified pave the way to therapeutic solutions and highlight the high heterogeneity of PCa with only these 5 PDX models, illustrated by our various pharmacological results. Different therapies can be tested based on identified genetic drivers, expression patterns and metabolic reprogramming. This information is critical for taking adapted treatment decision for patients, saving time and limiting side effects linked to inappropriate medication.

Overall, this collection spans the clinical heterogeneity of PCa including adenocarcinoma and neuroendocrine phenotypes. This is a dynamic repository, and, to date, we are constantly collecting samples to implement our PDX bank, allowing us to capture the evolving molecular landscape of PCa to support precision medicine.

### Limitations of our analyses

In accordance with the patient’s consent, no sequencing of the patient’s tumor or fragment of normal tissue were performed. This introduced a background noise in the calculation of the tumor mutational burden estimated between 100 to 200 somatic variants as it was performed based on a panel of normal and not patient’s tissue.

## Data availability statement

The datasets presented in this study can be found in online repositories. The names of the repository/repositories and accession number(s) can be found in the article/**Supplementary Material**.

## Ethics statement

The studies involving human participants were reviewed and approved by both Comité de Protection des Personnes Est IV and Comité de Protection des Personnes Sud-Ouest et Outre-mer, under approval numbers DC-2010-1193 and DC 2019-3565, respectively. Written informed consent was obtained from all participants for their participation in this study. The patients/participants provided their written informed consent to participate in this study. The animal study was reviewed and approved by Animal care and housing were conducted in accordance with the European Comity Council Directive 2010/63/UE and the French Ministry for Agriculture, Food and Forestry decree-2013. The study protocol was reviewed and approved by CEE-35 and CEEA-122 Ethical committee and approved by French Ministry for National Education, Higher Education and Research under approval numbers APAFIS#2949-2015113017594629v5 and APAFIS#14811-2018042316405732v6. Written informed consent was obtained from the individual(s) for the publication of any potentially identifiable images or data included in this article.

## Author contributions

YM had full access to all the data in the study and takes responsibility for the integrity of the data and the accuracy of the data analysis. CB, NB and ML designed, performed experiments or bioinformatics analyses, analyzed and interpreted the data. HL, CB and ML developed the PDXs models. HL and EP provided samples and helped with clinical data analysis. CB, NB and ML performed the pharmacological characterization of the PDXs. CB performed STR analysis. CK performed histo- and immunohistopathological analysis under YA supervision. YM and NB performed most of the bioinformatics analyses together with help JM-P for PDXs’ genomic and transcriptomic analysis. YM supervised CB, ML and NB. VL performed and supervised histopathological analysis of tumors and PDXs. YM and PL designed the study and supervised establishment of part of the PDXs and pharmacological characterization of PDXs. YM supervised the genomic analyses. TM and HL designed and supervised the establishment of part of the PDXs. YM designed and supervised the research, analyzed and interpreted the data. CB, NB and YM wrote the paper. All authors contributed to the article and approved the submitted version.

## Supplementary material

Supplementary Figure 1 Short Tandem repeat (STR) profiling of patient tumors and matched PDXs.

Supplementary Figure 2 Next-generation sequencing results. **(A)** Copy Number alterations (CNA) and B-Allele frequency of each PDX model are presented. First panel (CNA): Amplifications are depicted in green, gains in blue, losses in orange and deletions in purple. Copy-neutral loss of heterozygosity is represented in red compared to the reference in black. Second panel B-allele frequency is in red, and reference is in black. **(B)** The mutational burden for each PDX is represented by a green bar compared to the mutational load of reference tumors. The tumor mutational burden per megabase (TMB) is indicated in the log2 scale for each sample, and microsatellite instability–high (MSI-H) versus a microsatellite-stable (MSS) status. **(C)** Variant allele frequency comparison between C901 and C1022 PDX models. ​

Supplementary Figure 3 TMPRSS2 ERG fusion. Chromosome 21 representation for C901 and C1022 PDX models.

Supplementary Figure 4 Deciphering of CRPC-NE emergence through genomic analysis. Model of evolution occurring during prostate cancer progression for the patient from whom C901 and C1022 were generated, suggesting a common precursor and a divergent clonal evolution.

Supplementary Figure 5 Immunohistochemical staining for PDX model characterization. Representative immunochemical staining for NKX3.1, CD45 in PDX models. SYN (synaptophysin), and CgA (chromogranin) immunochemical staining in patient tumor and corresponding PDX model. Scale bar corresponds to 100 µm.

Supplementary Figure 6 Transcriptomic analysis. **(A)** Normalized enrichment score (NES) of GSEA for hallmark gene sets showing top up and down-regulated genes in metastatic tumors vs. localized tumors. **(B)** Expression of neuroendocrine markers in all PDX models. **(C)** Upregulated and downregulated metabolic hallmark pathways according to the AR level expression in PDX models.

Supplementary Figure 7 PDX response to docetaxel and enzalutamide. PDX response to docetaxel and enzalutamide treatment (n= 6-8 mice per group). Data represent the average tumor volume (mm^3^) of each group ± SEM. ns, P>0.05; *, P<0.05; **, P<0.01; ***, P<0.001; ****, P<0.0001 comparing treated to control groups using a two-way ANOVA followed by a Dunnet’s multiple comparisons post-test.​

Supplementary Table 1 Xenografted prostatic tissues.

Supplementary Table 2 Characteristics of the PCa PDX panel.

Supplementary Table 3 Immunohistochemical staining information.

Supplementary Table 4 Summary of clinical and pathological data of PCa patients whose tumor material was used to generate PDX models.

Supplementary Table 5 All somatic mutations.

Supplementary Table 6 All chromosome aberrations.

Supplementary Table 7 Amplifications and deletion.

Supplementary Table 8 List of differentially expressed genes.
