# Supplementary material for: A new tumorgraft panel to accelerate precision medicine in prostate cancer
Source: Front Oncol. 2023 May 26;13:1130048. doi: 10.3389/fonc.2023.1130048 (PMC10250751; doi:10.3389/fonc.2023.1130048)
Supplement: Supplementary Figure 1 — Short Tandem repeat (STR) profiling of patient tumors and matched PDXs. [file Presentation_1.pptx]

## Slide 1
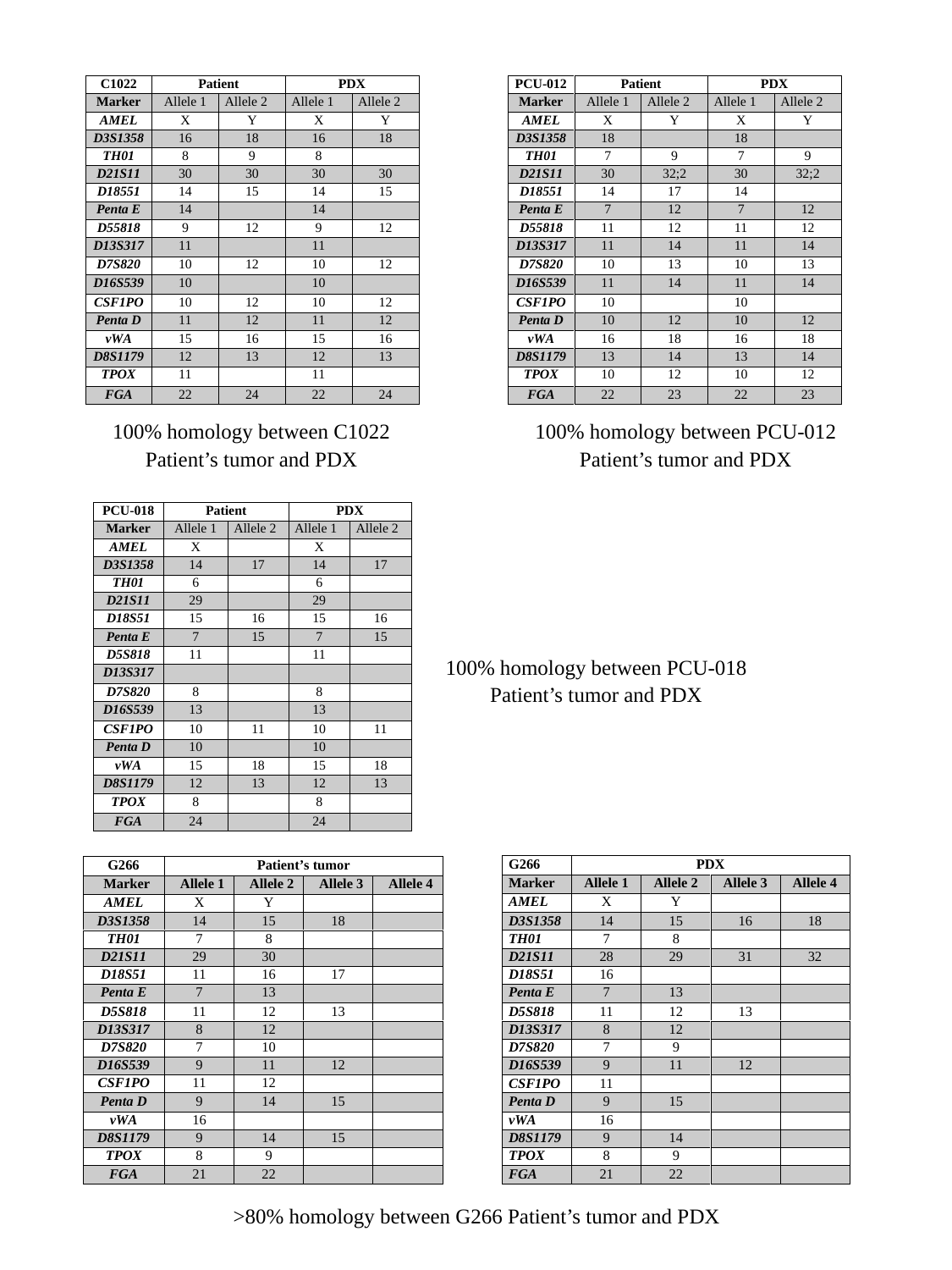

100% homology between C1022 Patient’s tumor and PDX
100% homology between PCU-012 Patient’s tumor and PDX
100% homology between PCU-018 Patient’s tumor and PDX
>80% homology between G266 Patient’s tumor and PDX
