# Supplementary material for: A new tumorgraft panel to accelerate precision medicine in prostate cancer
Source: Front Oncol. 2023 May 26;13:1130048. doi: 10.3389/fonc.2023.1130048 (PMC10250751; doi:10.3389/fonc.2023.1130048)
Supplement: Supplementary Figure 2 — Next-generation sequencing results. (A) Copy Number alterations (CNA) and B-Allele frequency of each PDX model are presented. First panel (CNA): Amplifications are depicted in green, gains in blue, losses in orange and deletions in purple. Copy-neutral loss of heterozygosity is represented in red compared to the reference in black. Second panel B-allele frequency is in red, and reference is in black. (B) The mutational burden for each PDX is represented by a green bar compared to the mutational load of reference tumors. The tumor mutational burden per megabase (TMB) is indicated in the log2 scale for each sample, and microsatellite instability–high (MSI-H) versus a microsatellite-stable (MSS) status. (C) Variant allele frequency comparison between C901 and C1022 PDX models. ​ [file Presentation_2.pptx]

## Slide 1
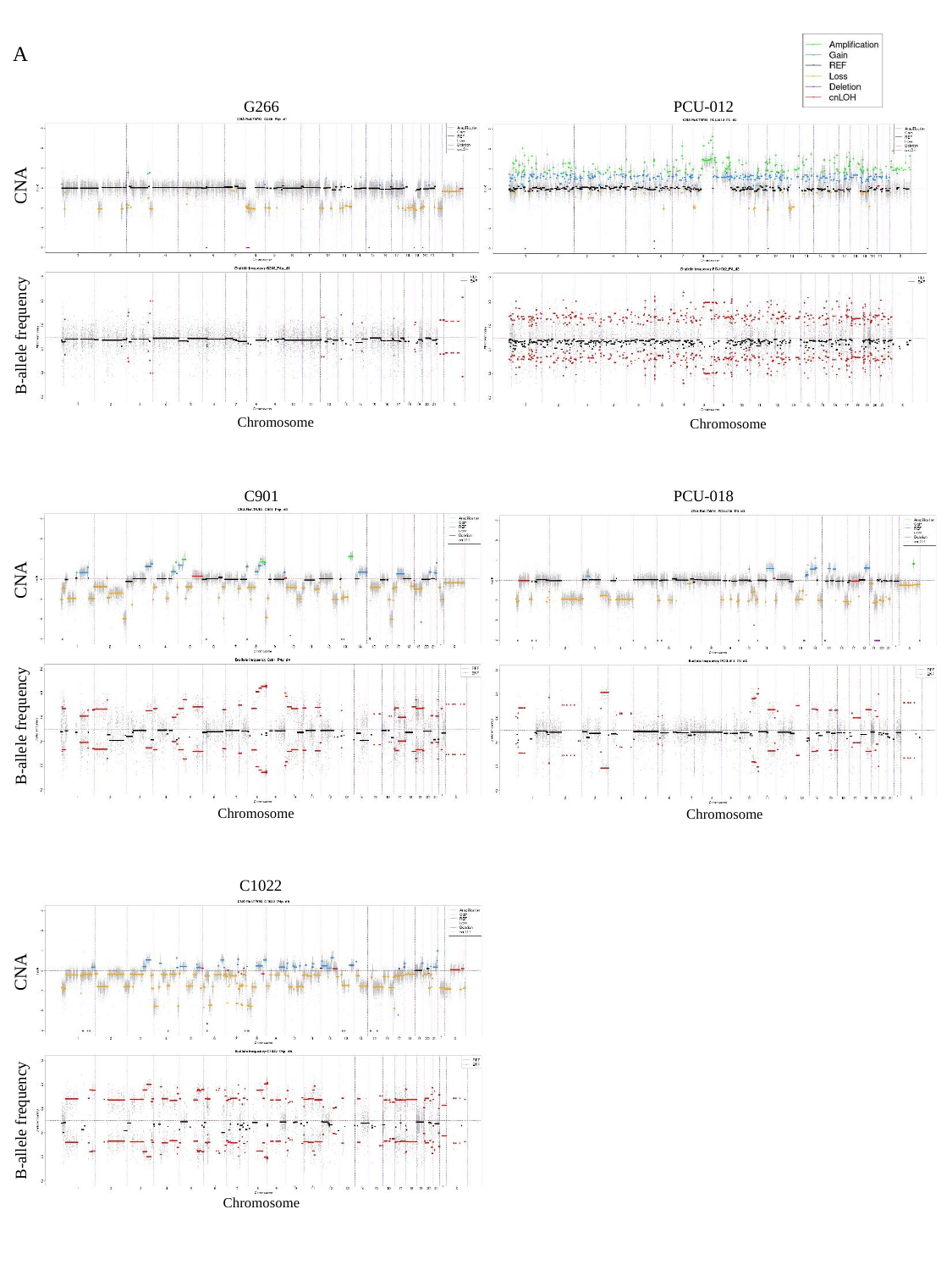

A
G266
PCU-012
CNA
B-allele frequency
Chromosome
Chromosome
C901
PCU-018
CNA
B-allele frequency
Chromosome
Chromosome
C1022
CNA
B-allele frequency
Chromosome

## Slide 2
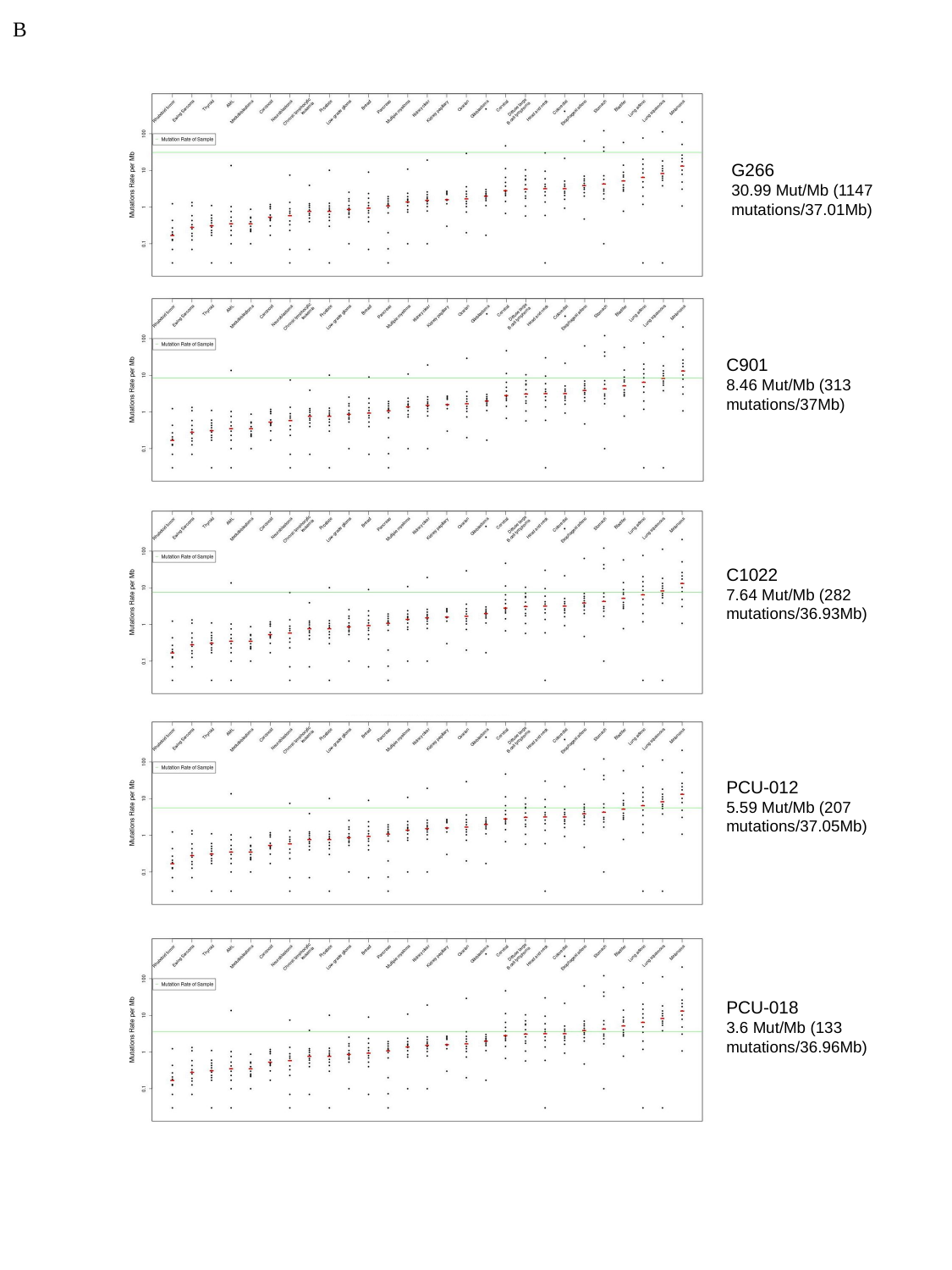

B
G266
30.99 Mut/Mb (1147 mutations/37.01Mb)
C901
8.46 Mut/Mb (313 mutations/37Mb)
C1022
7.64 Mut/Mb (282 mutations/36.93Mb)
PCU-012
5.59 Mut/Mb (207 mutations/37.05Mb)
PCU-018
3.6 Mut/Mb (133 mutations/36.96Mb)

## Slide 3
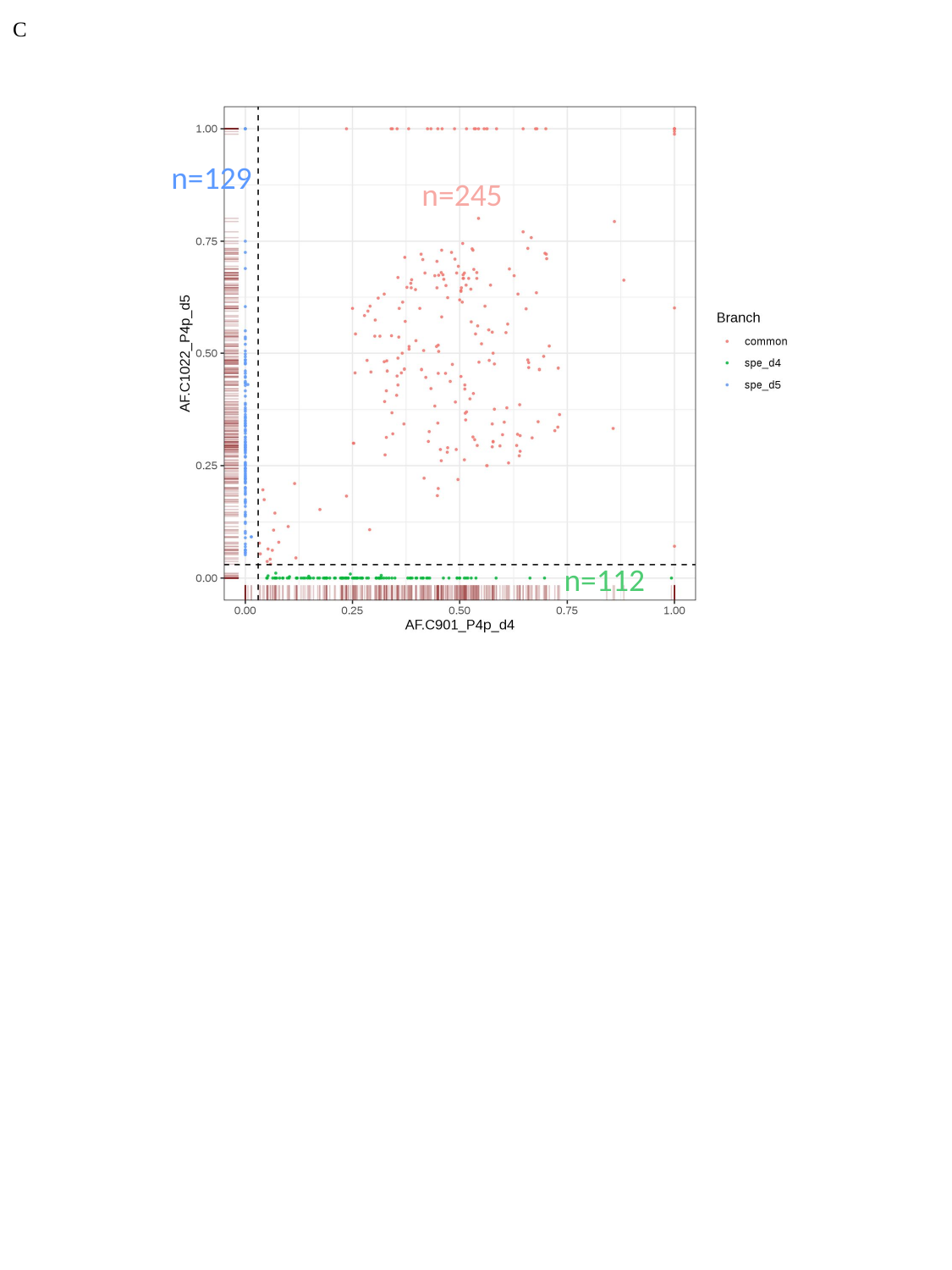

C
n=129
n=245
n=112
