# Supplementary figures and images for: A new tumorgraft panel to accelerate precision medicine in prostate cancer
Source: Front Oncol. 2023 May 26;13:1130048. doi: 10.3389/fonc.2023.1130048 (PMC10250751; doi:10.3389/fonc.2023.1130048)

## Slide 1
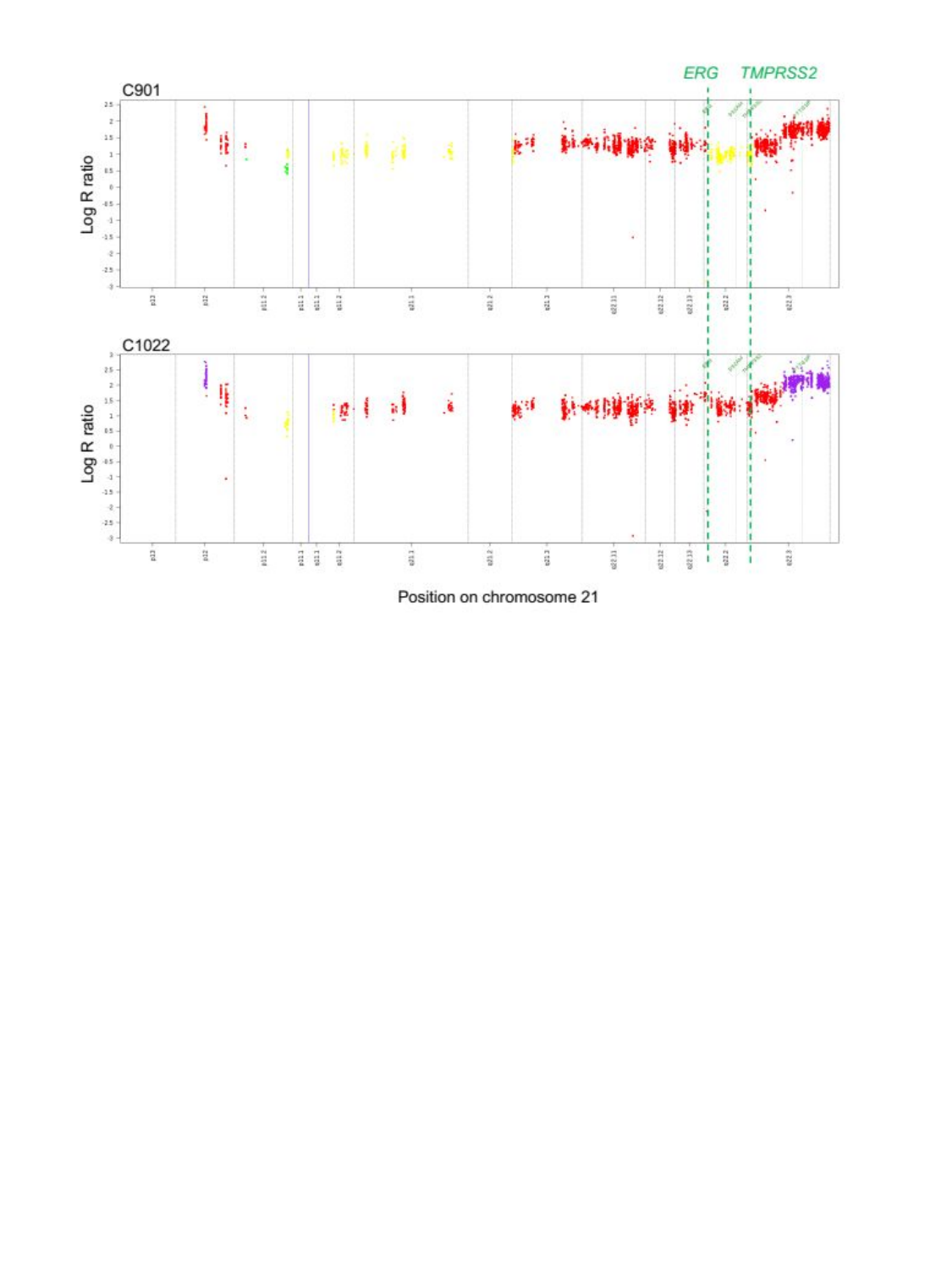

Supplement: Supplementary Figure 3 — TMPRSS2 ERG fusion. Chromosome 21 representation for C901 and C1022 PDX models. [file Presentation_3.pptx]

## Slide 1
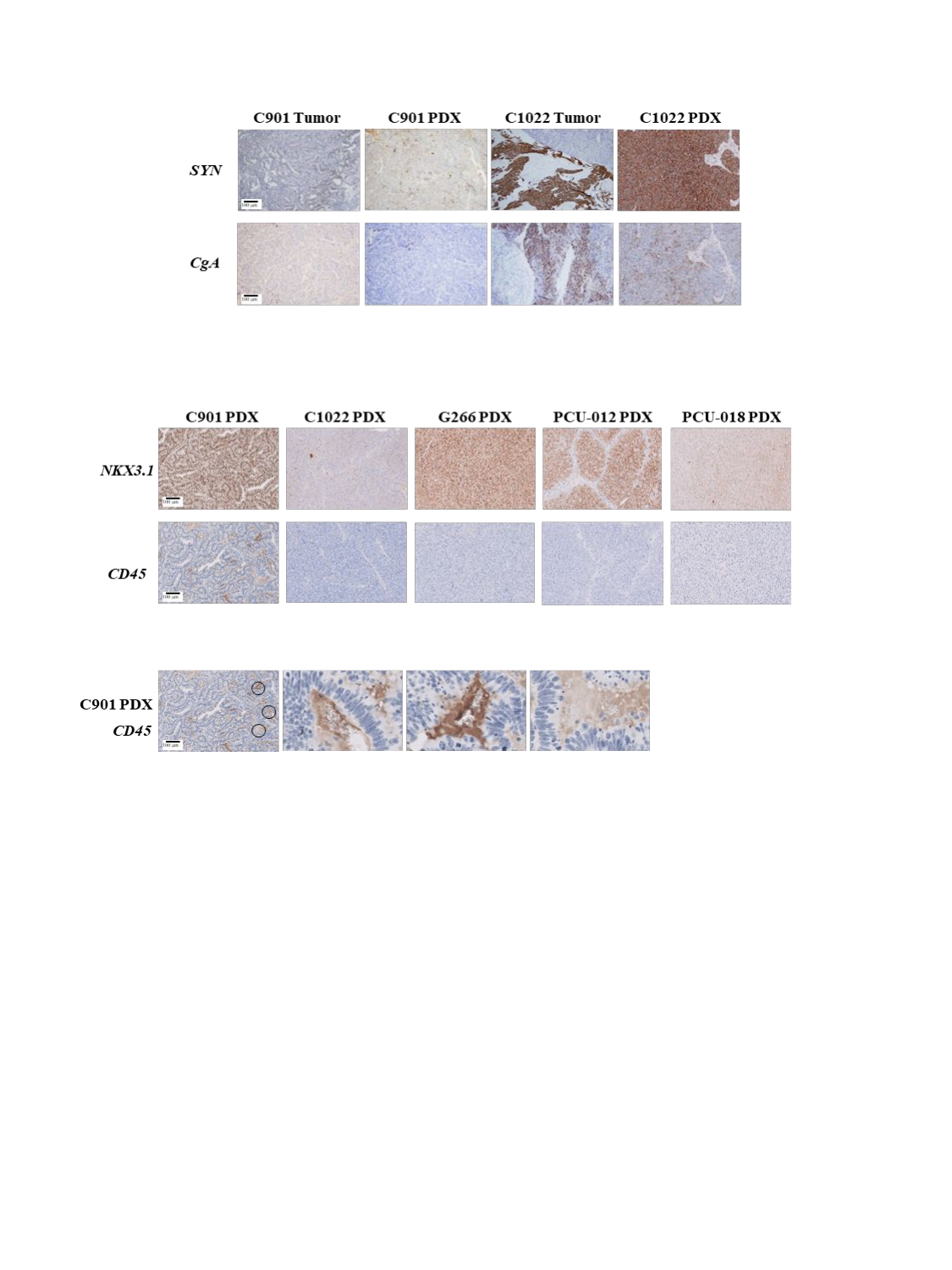

Supplement: Supplementary Figure 5 — Immunohistochemical staining for PDX model characterization. Representative immunochemical staining for NKX3.1, CD45 in PDX models. SYN (synaptophysin), and CgA (chromogranin) immunochemical staining in patient tumor and corresponding PDX model. Scale bar corresponds to 100 µm. [file Presentation_5.pptx]

## Slide 1
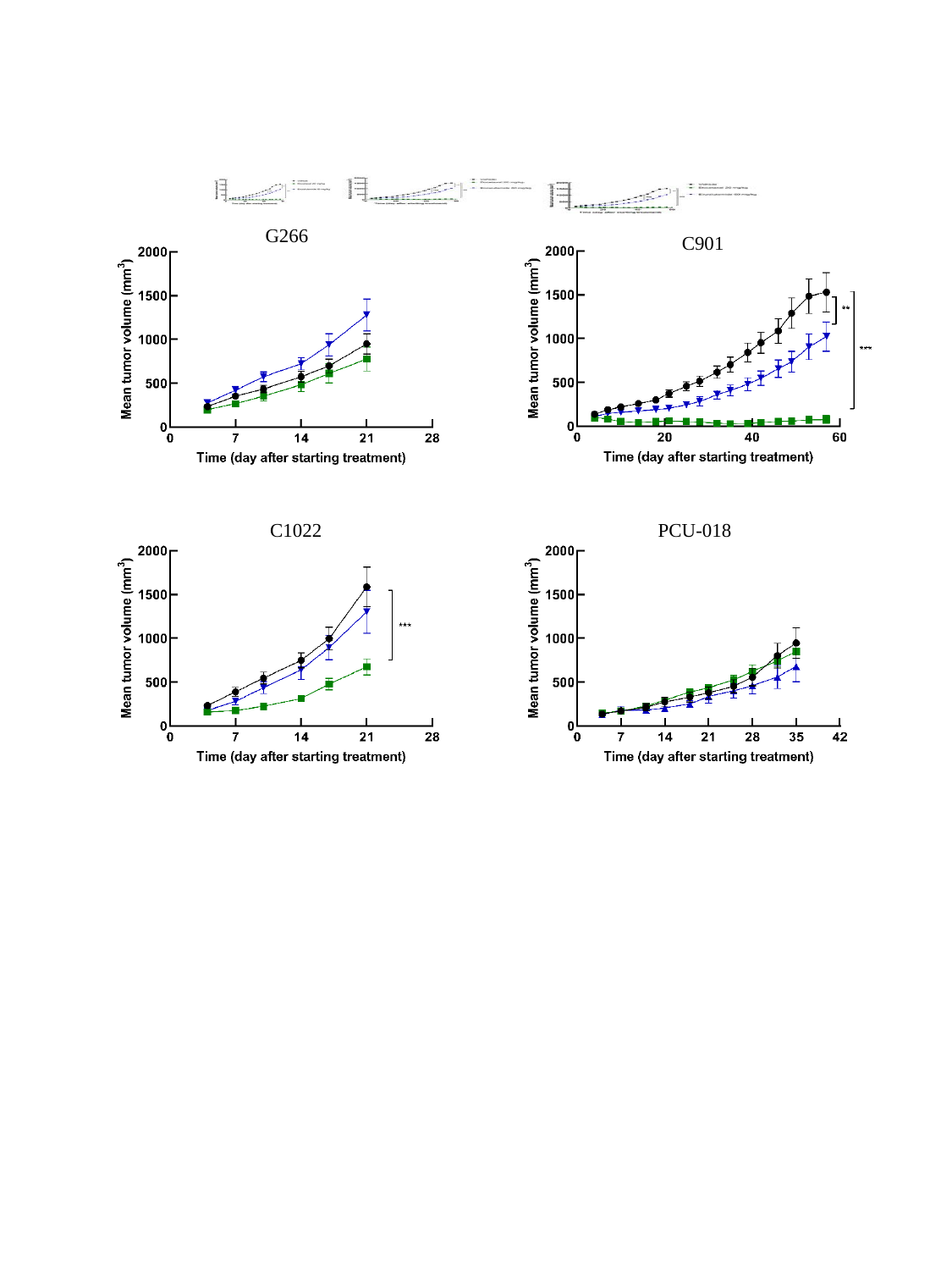

G266
C901
C1022
PCU-018

Supplement: Supplementary Figure 7 — PDX response to docetaxel and enzalutamide. PDX response to docetaxel and enzalutamide treatment (n= 6-8 mice per group). Data represent the average tumor volume (mm3) of each group ± SEM. ns, P>0.05; *, P<0.05; **, P<0.01; ***, P<0.001; ****, P<0.0001 comparing treated to control groups using a two-way ANOVA followed by a Dunnet’s multiple comparisons post-test.​ [file Presentation_7.pptx]
