# Supplementary material for: A new tumorgraft panel to accelerate precision medicine in prostate cancer
Source: Front Oncol. 2023 May 26;13:1130048. doi: 10.3389/fonc.2023.1130048 (PMC10250751; doi:10.3389/fonc.2023.1130048)
Supplement: Supplementary Figure 4 — Deciphering of CRPC-NE emergence through genomic analysis. Model of evolution occurring during prostate cancer progression for the patient from whom C901 and C1022 were generated, suggesting a common precursor and a divergent clonal evolution. [file Presentation_4.pptx]

## Slide 1
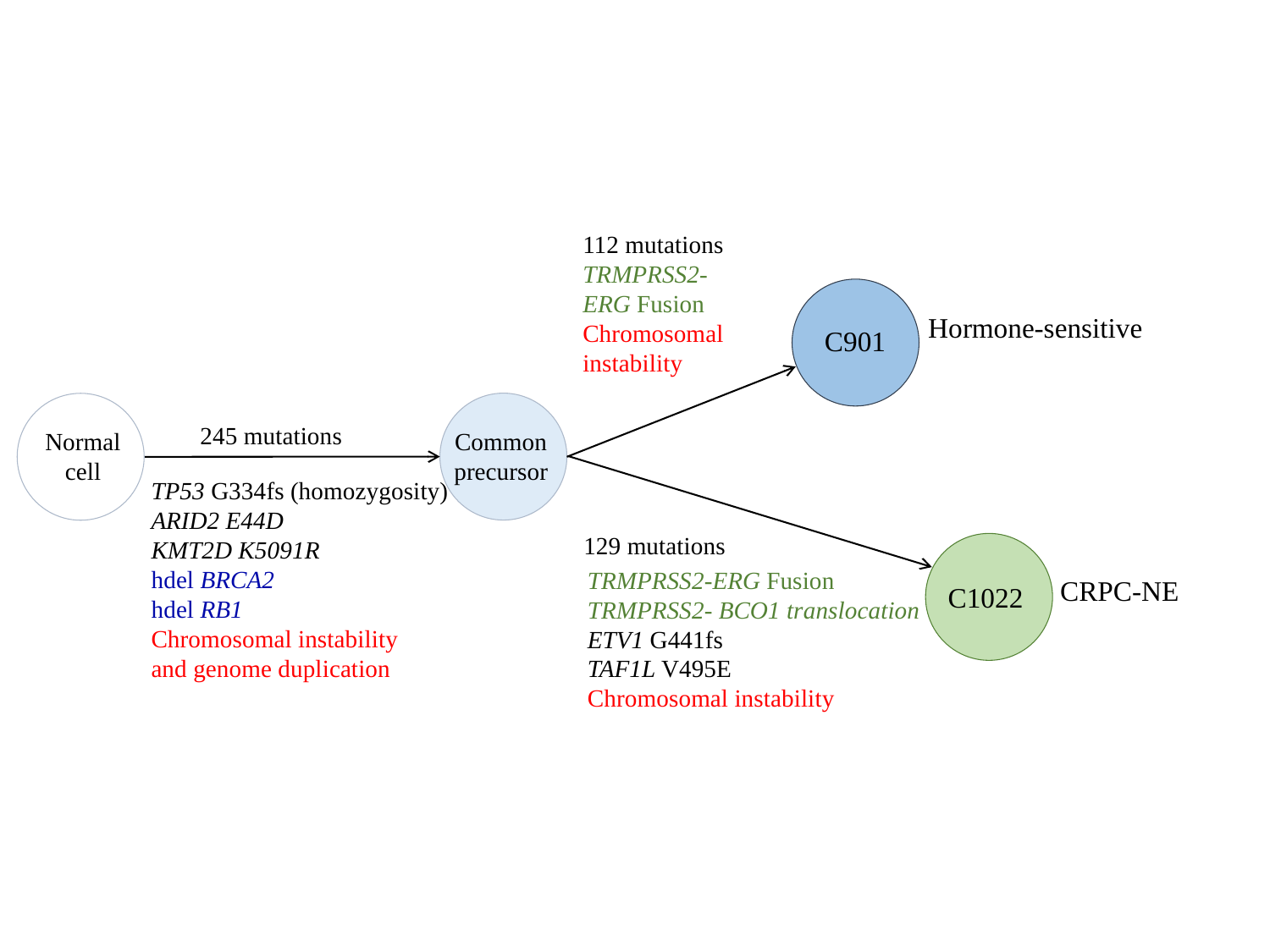

112 mutations
TRMPRSS2-ERG Fusion
Chromosomal instability
Hormone-sensitive
C901
245 mutations
Normal cell
Common precursor
TP53 G334fs (homozygosity)
ARID2 E44D
KMT2D K5091R
hdel BRCA2
hdel RB1
Chromosomal instability
and genome duplication
129 mutations
TRMPRSS2-ERG Fusion
TRMPRSS2- BCO1 translocation
ETV1 G441fs
TAF1L V495E
Chromosomal instability
CRPC-NE
C1022
