# Supplementary material for: A new tumorgraft panel to accelerate precision medicine in prostate cancer
Source: Front Oncol. 2023 May 26;13:1130048. doi: 10.3389/fonc.2023.1130048 (PMC10250751; doi:10.3389/fonc.2023.1130048)
Supplement: Supplementary Figure 6 — Transcriptomic analysis. (A) Normalized enrichment score (NES) of GSEA for hallmark gene sets showing top up and down-regulated genes in metastatic tumors vs. localized tumors. (B) Expression of neuroendocrine markers in all PDX models. (C) Upregulated and downregulated metabolic hallmark pathways according to the AR level expression in PDX models. [file Presentation_6.pptx]

## Slide 1
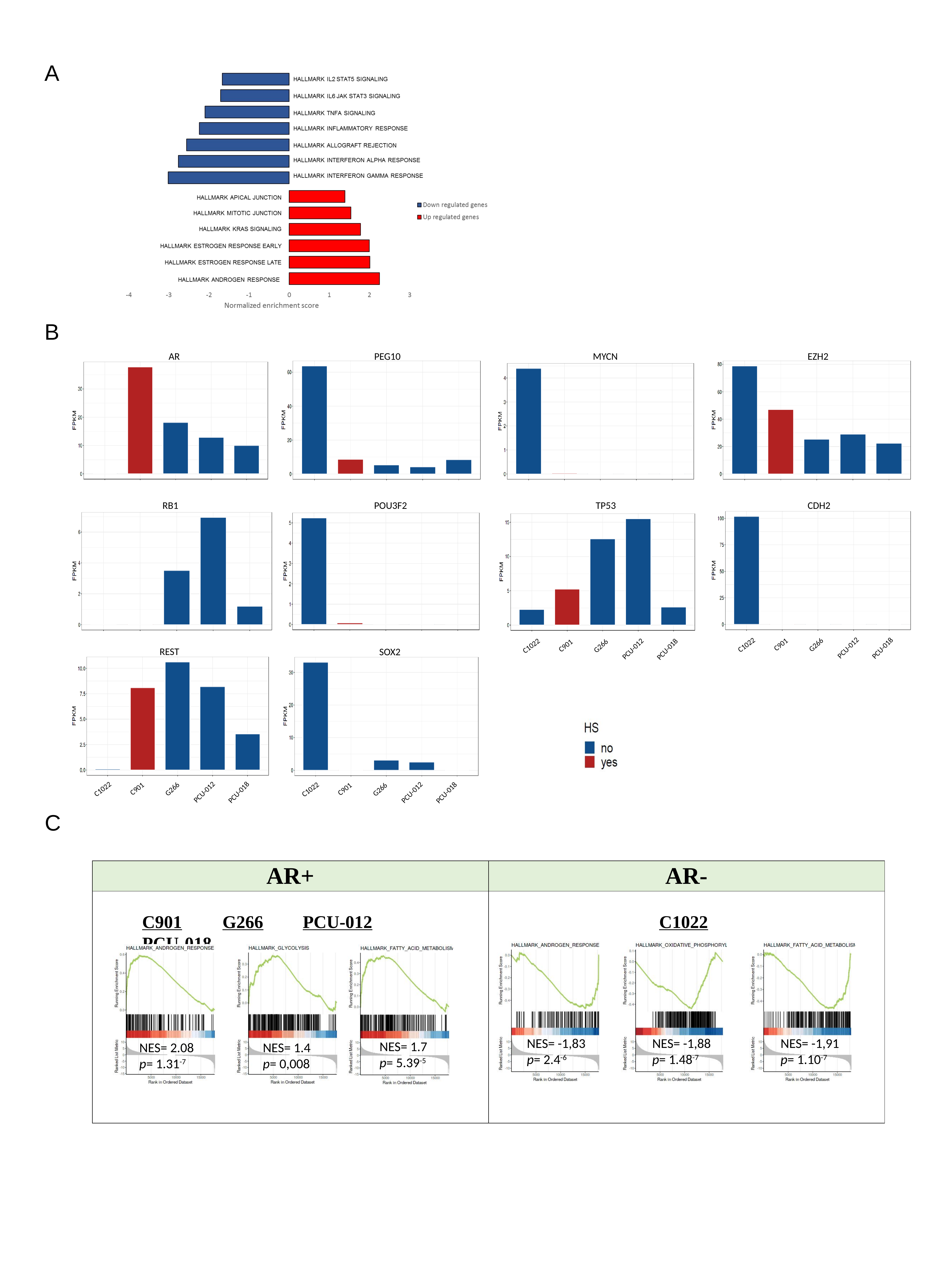

A
B
EZH2
MYCN
PEG10
AR
RB1
POU3F2
TP53
CDH2
C901
G266
C1022
PCU-012
PCU-018
C901
G266
C1022
PCU-012
PCU-018
REST
SOX2
C901
G266
C1022
PCU-012
PCU-018
C901
G266
C1022
PCU-012
PCU-018
C
| AR+ | AR- |
| --- | --- |
| | |
C1022
C901		G266	PCU-012		PCU-018
NES= -1,83
p= 2.4-6
NES= -1,88
p= 1.48-7
NES= -1,91
p= 1.10-7
NES= 1.7
p= 5.39-5
NES= 2.08
p= 1.31-7
NES= 1.4
p= 0,008
